# The desensitization gate of inhibitory Cys-loop receptors

**DOI:** 10.1038/ncomms7829

**Published:** 2015-04-20

**Authors:** Marc Gielen, Philip Thomas, Trevor G. Smart

**Affiliations:** 1 Department of Neuroscience, Physiology & Pharmacology, University College London, Gower Street, London WC1E 6BT, UK

## Abstract

Cys-loop neurotransmitter-gated ion channels are vital for communication throughout the nervous system. Following activation, these receptors enter into a desensitized state in which the ion channel shuts even though the neurotransmitter molecules remain bound. To date, the molecular determinants underlying this most fundamental property of Cys-loop receptors have remained elusive. Here we present a generic mechanism for the desensitization of Cys-loop GABA_A_ (GABA_A_Rs) and glycine receptors (GlyRs), which both mediate fast inhibitory synaptic transmission. Desensitization is regulated by interactions between the second and third transmembrane segments, which affect the ion channel lumen near its intracellular end. The GABA_A_R and GlyR pore blocker picrotoxin prevented desensitization, consistent with its deep channel-binding site overlapping a physical desensitization gate.

## Abstract (web summary)

Following activation, Cys-loop neurotransmitter-gated ion channels gradually desensitize and conductance shuts down. Here Gielen *et al.* present a mechanism for desensitization in which the interface between transmembrane domains is remodelled to constrict the channel pore at the intracellular end.

## Graphical abstract

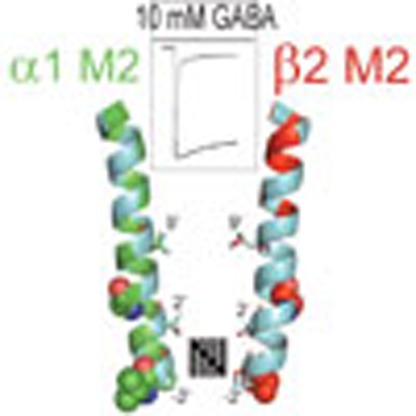

The presynaptic release of neurotransmitters is a prelude to their diffusion across the synaptic cleft and subsequent activation of postsynaptic ionotropic receptors, which are responsible for fast chemical neurotransmission. Agonist binding initiates the rapid opening of ligand-gated ion channels permitting a selective flow of ions through the pore. The sustained presence of the neurotransmitter will cause ligand-gated channels to transit from the active open-channel agonist-bound conformation to a desensitized shut-channel, agonist-bound state, thereby limiting current flow^1^,^2^. Receptor desensitization is a fundamental property of most ligand-gated ion channels and can have profound physiological consequences. These include the progressive reduction of postsynaptic current on repetitive synaptic neurotransmitter release^2^,^3^,^4^,^5^; the pre-desensitization of receptors by low ambient concentrations of neurotransmitter due to spillover from active neighbouring synapses^6^; and the slowing of synaptic current decays leading to their prolongation^5^.

In the central nervous system, neuronal activity results from a balance between excitation and inhibition. This is largely dictated by the activity of excitatory ionotropic glutamate receptors, and inhibition caused by GABA and glycine^7^ activating GABA_A_ (GABA_A_Rs) and glycine receptors (GlyRs). These latter receptors belong to the superfamily of pentameric Cys-loop receptors that also comprises excitatory nicotinic acetylcholine (nAChRs) and serotonin receptors (5HT_3_R)^7^. Differential desensitization kinetics of excitatory and inhibitory receptors has the potential to profoundly affect the spike firing profiles of neurons^5^ and thus neural network activity. At the molecular level, desensitization of AMPA and kainate classes of ionotropic GluRs is relatively well understood and involves structural rearrangements at the dimer interface between adjacent extracellular agonist-binding domains^8^,^9^,^10^,^11^. However, for the Cys-loop GABA_A_Rs and GlyRs, although the structure–function studies have improved our understanding of how these receptors activate^7^,^12^, the molecular determinants underlying the process of desensitization have yet to be defined.

Here we show that for the inhibitory neurotransmitter GABA_A_ and glycine receptors, the mechanism of agonist-induced desensitization is regulated by residue interactions between the transmembrane domains of the receptor, resulting in a time-dependent constriction of the ion channel pore that reduces the agonist-activated membrane conductance. This constriction has the characteristics of a desensitization ‘gate’ that is discrete from the ion channel gate involved in ligand-gated channel opening.

## Results

### Desensitization profiles differ between GABA_A_ receptors

**Figure 1:**
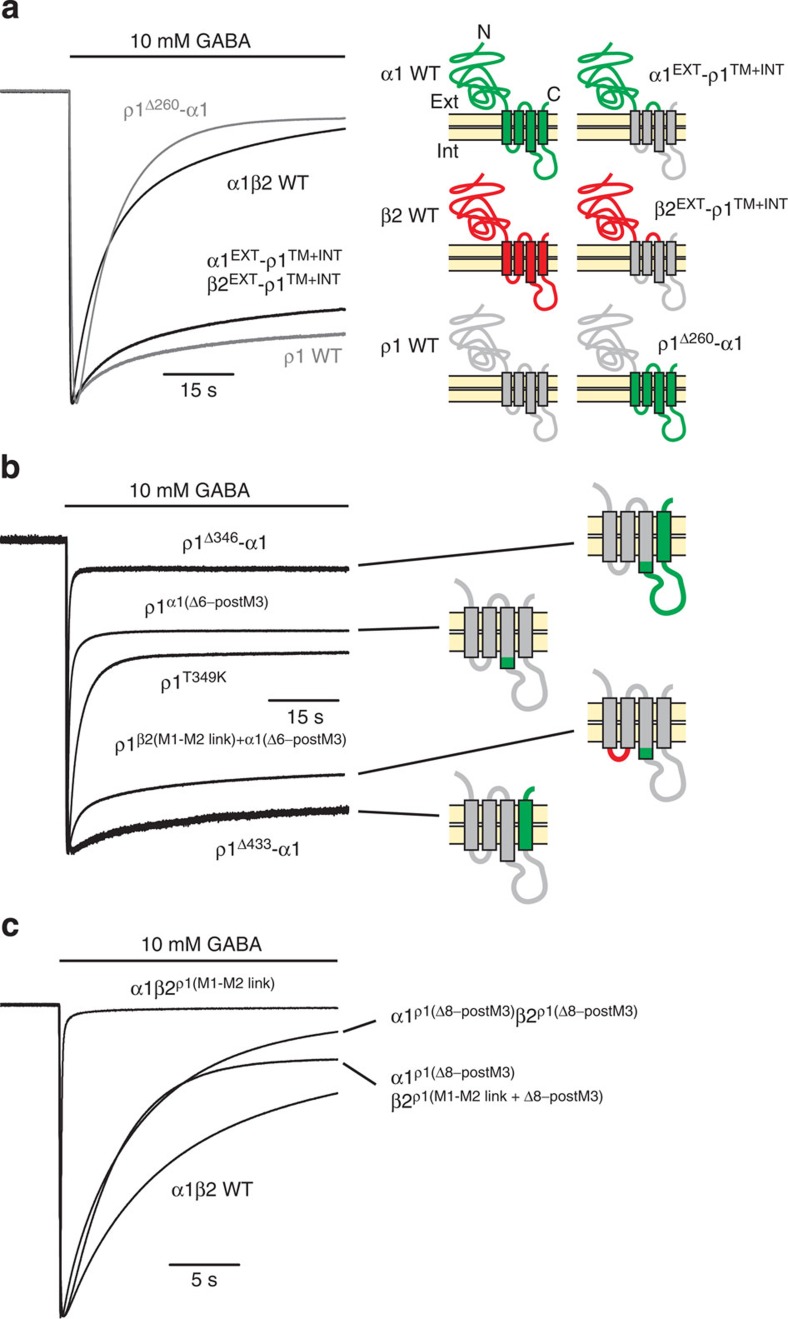
Intracellular end of M3 and the M1–M2 linker control desensitization of GABA_A_Rs. (**a**–**c**) Peak-scaled membrane currents elicited by 10 mM GABA (black line), showing the desensitization phase for the indicated receptor constructs (left column). The recovery phase is omitted for clarity. See Table 1 for values of *τ*_w_ and extent of desensitization (% Des) in all figures. The subunit chimeras are depicted by a colour code: green (α1), red (β2) and grey (ρ1) (right column). Numbering refers to the position of the interface between the two subunits in the chimera. TM/M, transmembrane domain, INT, intracellular loops.

To locate the molecular components responsible for desensitization, we selected two GABA_A_R isoforms that differ markedly in their response to supersaturating GABA concentrations (10 mM) by either desensitizing significantly (α1β2 heteromers; percentage of peak current desensitization (% Des)=86% and weighted decay time constant (*τ*_W_)=17 s) or minimally (ρ1 homomers, 19% Des and 23 s, Table 1, Fig. 1a). We then constructed chimeras to first explore the role of the extracellular domain (ECD) by replacing the ECD of α1 with the homologous section from ρ1 (ρ1^Δ260^-α1; Supplementary Fig. 1). The desensitization profile was similar to that observed for wild-type α1β2 receptors (Fig. 1a), consistent with published data^13^. Next we built chimeras containing M1 to M4 and the intracellular linkers (M1–M2 and M3–M4) from ρ1, coupled to the entire extracellular region of α1 or β2, corresponding to their ECDs and the external M2–M3 linkers (chimeras α1^EXT^-ρ1^TM+INT^ and β2^EXT^-ρ1^TM+INT^, respectively; Supplementary Fig. 1). Co-expression of these chimeras supported large GABA currents with limited desensitization, comparable to the profile for wild-type ρ1 (Fig. 1a, see Table 1 for all time constants and extents of desensitiszation data and Supplementary Table 1 for GABA current amplitudes), thereby locating the main determinants of desensitization to the transmembrane and/or intracellular regions of the receptor.

Our primary measure of desensitization involved monitoring the decline of currents activated by maximal saturating concentrations of GABA. To ensure that the mutations did not adversely perturb the general structure, function or long-term trafficking of the receptor, we monitored both the dose–response curves and the recovery phase of the currents from desensitization for selected mutants. Overall, the concentration–response curves and EC_50_ values were minimally affected by the mutations, though certain GABA receptor constructs did generate slightly lower EC_50_ values, in accord with an increase in affinity that is often associated with desensitized receptors (Supplementary Fig. 2). Recoveries of the maximal peak GABA currents following desensitization were also similar for wild-type and singly mutated receptors, with complete recovery from maximal desensitization always achieved in mins (Supplementary Fig. 3). In addition, we have reported both the rates and extent of desensitization for all mutants to gain an overall view of the desensitization process (Table 1).

### Internal end of M3 and M1–M2 linker control desensitization

We refined our search using homomeric ρ1 receptors and sequentially replacing individual transmembrane segments, M1 to M3, with those from α1. These constructs yielded very small currents, precluding analysis; however, one chimera incorporating the intracellular end of M3, the M3–M4 intracellular linker and M4 of α1 into the ρ1 subunit (ρ1^Δ346^-α1), produced robust currents, which strongly desensitized (%Des=94% and *τ*_W_=200 ms, Fig. 1b).

Surprisingly, incorporating just M4 from the α1 subunit did not affect the desensitizing phenotype of ρ1 (ρ1^Δ433^-α1, Fig. 1b). We therefore targeted M3 in ρ1 and replaced six contiguous residues at the carboxyl-terminal (C-terminal; intracellular) end of M3 with the homologous residues from α1. This produced a receptor (ρ1^α1(Δ6-postM3)^) that desensitized profoundly within one second following activation by GABA (Fig. 1b). Within this six amino-acid cassette, substituting just one residue, ρ1^T349K^, was sufficient to confer a rapid desensitizing profile on ρ1 receptors (Fig. 1b).

Interestingly, on the ρ1^α1(Δ6−postM3)^ background, incorporating the M1–M2 intracellular linker from the β2 subunit considerably reduced desensitization towards that for wild-type ρ1 (chimera ρ1^β2(M1–M2 link)+^^α1(Δ6-postM3)^, Fig. 1b). Such a reversion was not observed after incorporating the α1 M1–M2 linker (Supplementary Fig. 4). These data indicate that the intracellular end of M3 and the M1–M2 linker of β2 modulate desensitization, conceivably via an intersubunit (α–β) interaction, which is consistent with the close proximity of α1 M3 C-terminal end to the M1–M2 linker of an adjacent β2 subunit, as seen in our structural models (see below).

We subsequently examined this potential interaction in α1β2 GABA_A_Rs by first introducing the M1–M2 linker of ρ1 into β2 subunits (β2^ρ1(M1–M2 link)^). Co-expressing this chimera with wild-type α1 produced receptors that, unexpectedly, desensitized almost completely at a rate 50-fold faster than wild-type α1β2 receptors (Fig. 1c). Moreover, exchanging eight residues from the intracellular end of M3 in α1 and β2 with those from ρ1 resulted in a modest three-fold increase in the desensitization rate for α1^ρ1(Δ8-postM3)^β2^ρ1(Δ8-postM3)^ (Fig. 1c). However, on this background, reintroducing the M1–M2 linker of ρ1 into β2 (α1^ρ1(Δ8-postM3)^β2^ρ1(M1–M2 link+Δ8-postM3)^, produced relatively little effect on desensitization compared with the wild-type receptor (Fig. 1c). If the combined effect of these two modifications had been additive, we would have expected the double chimera to desensitize even faster than the α1β2^ρ1(M1–M2 link)^ receptors. Therefore, the non-additive nature of these mutations further supports an interaction between the intracellular end of M3 and the M1–M2 linker, which could represent a cassette of complementary residues conferring desensitizing properties on the GABA ion channel.

### M2 and M3 interface strongly affects GABA_A_R desensitization

**Figure 2:**
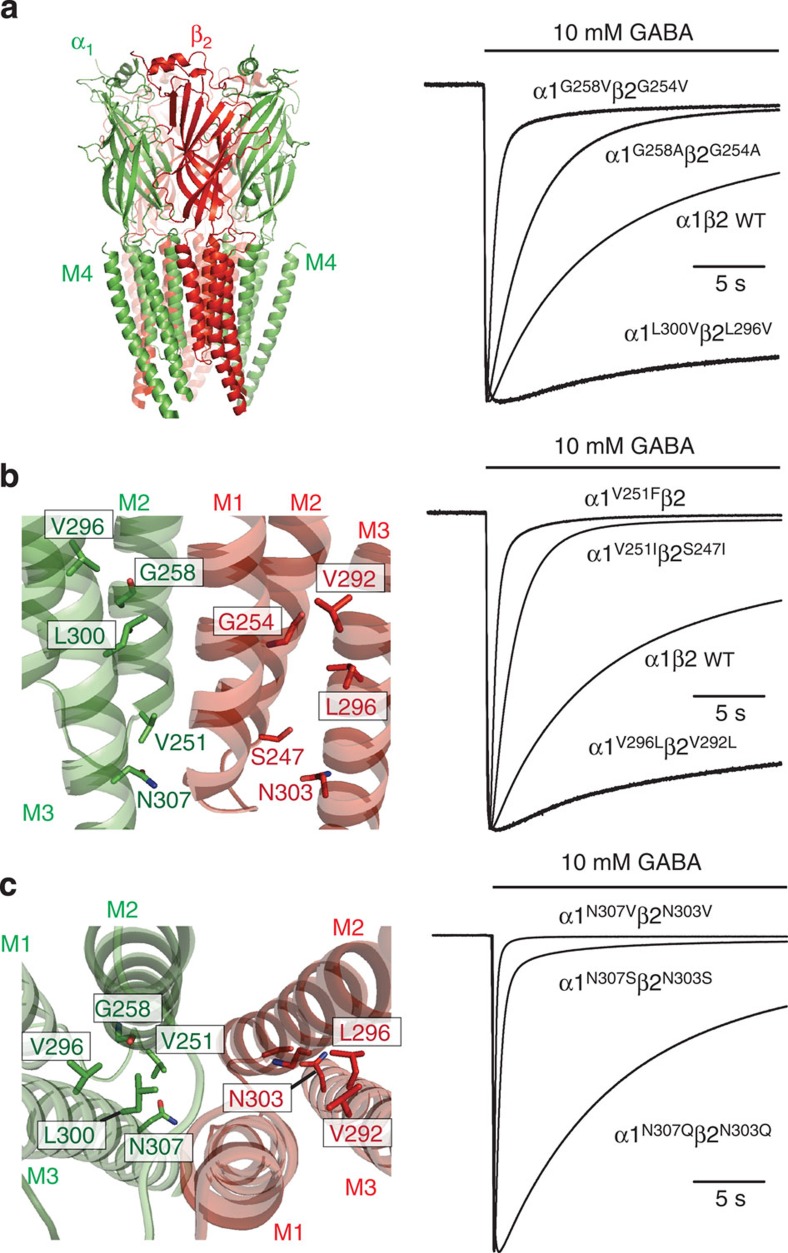
Mutating the intracellular end of the M2/M3 interface regulates desensitization of α1β2 GABA_A_Rs. (**a**) Left: 3D model of an α1β2 GABA_A_R based on GluCl template. Right in **a**–**c**: membrane currents induced by 10 mM GABA. (**b**) Left: enlarged side view of the intracellular end of the ion channel showing transmembrane domains M1–M3 for α1 (green) and β2 (red) subunits and the positions of various labelled residues. (**c**) Left: enlarged plan view of the ion channel. Note, in **b**,**c**, M4 segments were omitted for clarity.

**Figure 3:**
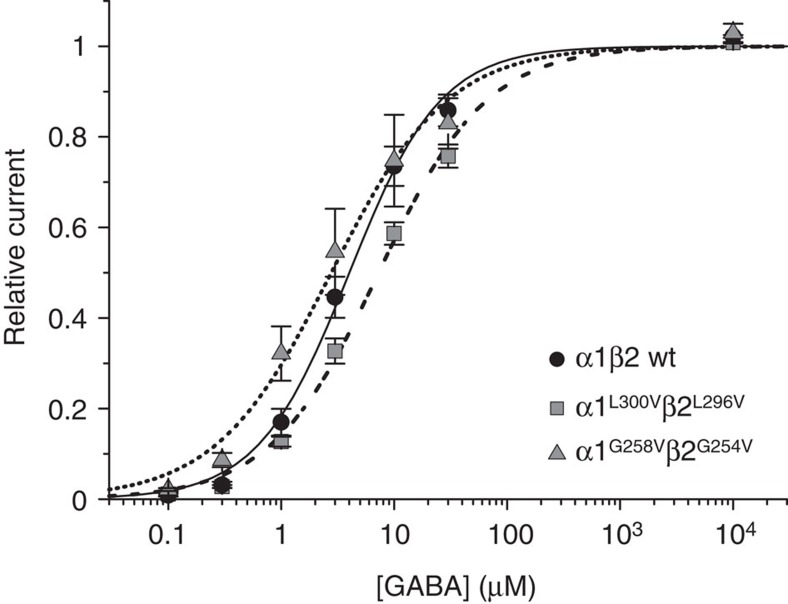
Effects of mutations α1^L300V^β2^L296V^ and α1^G258V^ β2^G254V^ on GABA sensitivity. GABA concentration–response curves for wild-type (wt) α1β2 (EC_50_=4.1±0.7 μM, *n*_H_=1.08±0.07, *n*=6), α1^L300V^β2^L296V^ (EC_50_=7.3±0.9 μM, *n*_H_=0.91±0.02, *n*=5) and α1^G258V^ β2^G254V^ receptors (EC_50_=3.0±0.9 μM, *n*_H_=0.89±0.19, *n*=5). Error bars are s.d.

While the identified cassette critically modulates desensitization, it is not the sole determinant. Indeed, exchanging the entire transmembrane portion of M3 for α1 and β2 with ρ1, virtually eliminated desensitization when these two chimeric subunits were co-expressed (α1^ρ1M3^β2^ρ1M3^, Supplementary Fig. 5). We therefore compared the primary sequences for M3, and noted that the residue homologous to V338 in ρ1 is a conserved leucine in other GABA_A_R and GlyR subunits (Supplementary Fig. 6). By substituting these leucines for valines (α1^L300V^β2^L296V^), we created receptors that displayed very little desensitization (Fig. 2a). Notably, this was achieved with very little change in the GABA concentration–response curve for α1^L300V^β2^L296V^ compared with that for wild-type receptors (Fig. 3), indicating the gating efficacy was minimally affected.

To understand how M3 residues could affect receptor function, we constructed a three-dimensional (3D) model of the α1β2 GABA_A_R (Fig. 2a) based on the structure of the *C. elegans* glutamate-gated Cl^−^ channel (GluCl) in the open conformation^14^, which shares high sequence homology with GABA_A_Rs and GlyRs (Supplementary Fig. 6). In this model, the side chain of the M3 leucine (α1^L300^) is orientated towards M2 near the 4′ position (Fig. 2b,c; Supplementary Fig. 7), and therefore any conformational changes to M3 may be detected in M2 by a proximal glycine residue (α1^G258^) that is highly conserved in all GABA_A_R and GlyR subunits (Supplementary Fig. 6).

Replacing this conserved glycine with neutral alanine in α1 and the β2 subunits (α1^G258A^β2^G254A^) produced receptors that desensitized 2.5-fold faster than the wild type, while mutation to bulky hydrophobic valines (α1^G258V^β2^G254V^) increased the desensitization rate by 14-fold (Fig. 2a) without changing the GABA concentration–response curve (Fig. 3).

We next assessed the importance of the highly conserved M3 valine (α1^V296^), which is located one α-helical turn above α1^L300^ (Supplementary Fig. 6). Together these residues effectively straddle the 4′ glycine (G258) in M2 (Fig. 2b). Significantly, their mutation to leucines in α1^V296L^β2^V292L^ strongly disrupted desensitization, further suggesting that rearrangements to the lower part of the M2/M3 intrasubunit interface underlies the process of receptor desensitization (Fig. 2b).

Exploring this interface further, we examined the consequences of mutating α1^V251^ and α1^N307^. Valine 251 is located at the −3′ position intracellular to M2, bordering the M1–M2 linker, and facing M3 at the level of α1^N307^ (Fig. 2b,c). Mutating α1^V251^ and the homologous β2^S247^ to isoleucines (Fig. 2b), alanines or aspartates (Table 1), produced a 7- to 14-fold increase in the rate of desensitization, while phenylalanine mutants (α1^V251F^β2^S247F^) did not yield a resolvable current; however, the co-expression with their wild-type counterparts (α1^V251F^β2 and α1β2^S247F^) created receptors desensitizing more than 20-fold faster than wild-type α1β2 counterparts (Fig. 2b, Table 1). It is possible that the double mutant receptors are functional but may desensitize too rapidly to be resolved. Indeed, we used excised patch recordings (see Methods) but still failed to resolve any current, which may reflect a desensitization rate that is faster than the rate of ion channel opening.

Similar results were obtained with the adjacent residue α1^N307^ and the homologous β2^N303^. Conservatively exchanging these asparagines for glutamines did not affect receptor function, while α1^N307S^β2^N303S^ and α1^N307V^β2^N303V^ receptors desensitized 15- and 100-fold faster, respectively, compared with wild-type α1β2 (Fig. 2c, Table 1). Co-expression of α1^N307D^ and β2^N303D^ failed to elicit current, but co-expression with their respective wild-type subunits revealed 4- to 40-fold increases in desensitization kinetics (Table 1). The lack of current, apparent with these double mutants, could thus be due to their profoundly enhanced rate of desensitization. Overall these results are in complete accord with residues located at the M2/M3 interface in the lower part of the membrane, playing a key role in receptor desensitization.

To investigate the importance of some of the key residues at the M2–M3 interface, and to ensure that the use of *Xenopus* oocytes was not altering the desensitizing profiles of the GABA-activated currents, we explored the impact of several receptor mutants when expressed in HEK cells. As observed with the oocyte-based experiments, the expression of α1^V251F^β2 generated currents that desensitized faster, by ∼10-fold compared with those for the α1β2 wild type (Supplementary Fig. 8, Supplementary Table 3). Again, similar current profiles were evident by co-expressing the nearby residue, α1^N307V^ with the homologous β2^N303V^, in HEK cells giving a sevenfold increase in the desensitization rate (Supplementary Fig. 8, Supplementary Table 3).

### γ2 subunit M2 and M3 affects α1β2γ2 GABA_A_R desensitization

**Figure 4:**
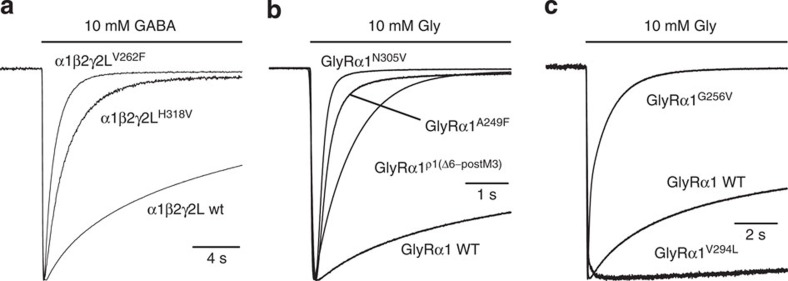
Residues affecting desensitization in heteromeric α1β2γ2 GABA_A_ receptors and homomeric GlyRα1 glycine receptors. (**a**) Membrane currents activated by 10 mM GABA showing desensitization of γ2 subunit-containing wild-type and mutant GABA_A_ receptors. (**b**,**c**) Membrane currents activated by 10 mM glycine, showing desensitization of glycine receptors, both wild type and mutants (see text for details).

To initially simplify our structural approach to exploring desensitization, we used α1β2 GABA_A_Rs. However, native synaptic GABA_A_ receptors will contain a single copy of the γ2 subunit^15^. To probe desensitization in the αβγ receptor, we examined the effect of mutations at the intracellular end of the γ2 subunit. We selected γ2^V262^ and γ2^H318^ for mutation since these are homologous to α1^V251^ and α1^N307^, respectively, which have a profound effect on desensitization of αβ receptors (Supplementary Fig. 6). While co-expressing wild-type α1, β2 and γ2L subunits produced receptors that desensitized similarly (% Des=80%, *τ*_W_=15.5 s) to wild-type α1β2, mutating γ2L^V262F^ and γ2L^H318V^ increased the rate of desensitization by 12- and 7-fold respectively, and strongly increased the extent of desensitization (% Des=98.7 and 96.3, respectively; Fig. 4a). These mutations did not generally perturb receptor function as GABA potency was minimally affected at α1β2γ2L^V262F^ and α1β2γ2L^H318V^ compared with wild-type α1β2γ2L GABA_A_Rs (Supplementary Fig. 9), suggesting that receptor gating was unaffected. Moreover, we also examined another key pharmacological facet of γ2-containing GABA_A_Rs involving modulation by benzodiazepines^16^. Diazepam-induced potentiation of low concentration (EC_1-2_, 1–3 μM) GABA currents at wild-type α1β2γ2L, and mutant α1β2γ2L^V262F^ and α1β2γ2L^H318V^ receptors, remained unaltered (2.53±0.20-fold (α1β2γ2L); 2.14±0.16-fold (α1β2γ2L^V262F^); and 2.51±0.05-fold (α1β2γ2L^H318V^), *n*=3), again suggesting that the mutations have neither affected gating, nor assembly, nor the folding of the receptors. It is important to note that, since there is only one copy of the γ2 subunit per α1β2γ2 receptor pentamer, there is also only one copy of the γ2 subunit mutation, further emphasizing the importance of the cytoplasmic end of the M2/M3 interface on the desensitization of Cys-loop receptors.

### A similar mechanism accounts for the desensitization of GlyRs

If the identified residues in GABA_A_Rs form a part of the generic mechanism for receptor desensitization at inhibitory Cys-loop receptors, then we should be able to replicate the effects in GlyRs. Applying saturating concentrations (10 mM) of glycine to GlyR α1 homomeric receptors (GlyRα1) evoked peak currents that desensitized by ∼72% with a *τ*_w_ of 11 s (Table 1, Fig. 4b,c). As mutations to the M1–M2 linker of GlyRα1 have been reported to strongly affect desensitization^17^, we focused on the intracellular end of M3, as we had done with the GABA_A_R, and exchanged six consecutive residues with those from ρ1. This chimera (GlyRα1^ρ1(Δ6-postM3)^) desensitized by ∼98% (*τ*_w_=0.9 s, Fig. 4b), establishing the intracellular end of M3 in GlyRs as a key component for desensitization.

We then examined the effect of single mutations towards the cytoplasmic end of M2 and M3. GlyRα1^L298^ is the homologous M3 leucine to GABA_A_R α1^L300^. Although the mutant GlyRα1^L298V^ did not affect desensitization (Table 1), mutating the highly conserved M2 glycine in GlyRα1^G256V^, significantly increased desensitization (% Des=99.4%, *τ*_W_=0.8 s, Fig. 4c) while minimally affecting the apparent affinity for glycine (Supplementary Fig. 2b). Furthermore, mutating the M3 V294 (GlyRα1^V294L^; homologous to GABA_A_R α1^V296^) virtually abolished desensitization (Fig. 4c). At the intracellular end of M2 and M3, we then targeted GlyRα1^A249^ (M2) and GlyRα1^N305^ (M3), which are homologous to GABA_A_R α1^V251^ and α1^N307^. In accord with our GABA_A_R data, the mutant GlyRα1^A249F^ and GlyRα1^N305V^ displayed near-complete desensitization with rates 13- and 60-fold higher, respectively, than wild-type GlyRα1 (Fig. 4b). This is consistent with our results for α1β2 GABA_A_Rs, and indicates that similar determinants and therefore mechanisms of desensitization apply to both GABA_A_Rs and GlyRs, involving structural rearrangements at the cytoplasmic end of the M2/M3 interface.

### Locating the desensitization gate using a channel blocker

**Figure 5:**
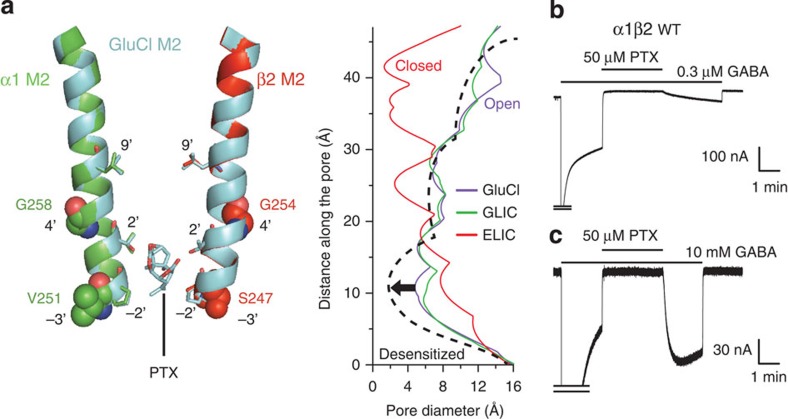
Promoting desensitization of GABA_A_Rs causes fast dissociation of the pore blocker PTX. (**a**) Left: side-view cutaway section through the transmembrane domains showing two (α1—green, β2—red) of the five M2 helices from our α1β2 GABA_A_R model overlaid with the M2 helices of the PTX-bound GluCl structure (light blue; pdb 3RI5 (ref. 14)). Note the deep location of the PTX-binding site between locations 2′ and −2′. Right: predicted pore diameter plotted as a function of the distance along the pore axis for GluCl, GLIC (open; pdb 3EAM (ref. 18)) and ELIC (shut; pdb 2VL0 (ref. 19)). This is vertically aligned with the left panel schema for cross comparison. The dashed line reflects the hypothesis that desensitization is an extension of the activation process, leading to a desensitization gate located between positions −3′ and 4′. (**b**) Representative membrane currents showing slow dissociation of PTX from α1β2 GABA_A_Rs when activated by a low concentration of GABA (0.3 μM, *n*=6). (**c**) Membrane currents showing fast dissociation of PTX from α1β2 GABA_A_Rs activated by saturating concentrations of GABA (10 mM, *n*=5). Note the peak currents are truncated for clarity.

To understand how the identified residues regulate the desensitization in GABA_A_Rs and GlyRs, we compared the crystal structure of GluCl, with those of ELIC and GLIC, the prokaryotic homologues of Cys-loop receptors in putative shut and open conformations, respectively^18^,^19^,^20^. While receptor activation involves an increase of the pore diameter between the 9′ and 20′ positions, there is a corresponding constriction of the pore at its cytoplasmic end, between −3′ and 4′ (Fig. 5a)^14^,^18^. We hypothesized that desensitization could be an extension of the conformational rearrangements that occur during the activation process, which is consistent with desensitization proceeding from the open state of the channel^21^,^22^. This implies that desensitization would involve a gate that is located proximal to the cytoplasmic end of the channel.

**Figure 6:**
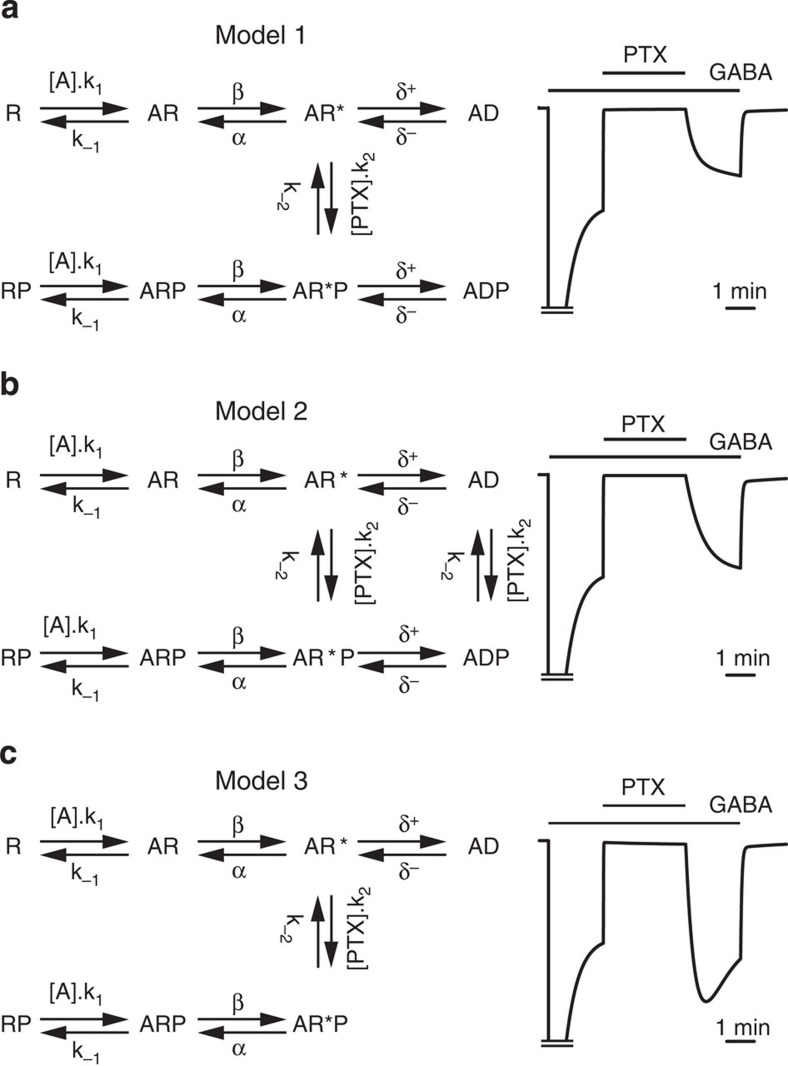
Simulation of GABA currents after block by and recovery from PTX. (**a**–**c**) Left: kinetic models 1–3 used to predict the recovery of agonist-induced currents after wash-out of PTX (or P) in the presence of saturating concentrations of the agonist ([A]; 10 mM GABA). Receptors states are: R inactive, agonist-unbound receptor; AR inactive, agonist-bound receptor; AR* agonist-bound activated receptor; AD agonist-bound desensitized receptor, all with or without bound PTX. Right: predicted membrane currents, generated from the models, for wild-type α1β2 GABA_A_Rs activated by 10 mM GABA in the presence and absence of 50 μM PTX. See Supplementary Table 2 for the numerical values of the parameters. (**a**) Model 1: the PTX-blocked open receptor (AR*P) can desensitize, but PTX binding and unbinding cannot occur to or from either the resting (R/RP, AR/ARP) or desensitized (AD/ADP) receptor states. (**b**) Model 2: PTX binding and unbinding is permitted to both open (AR*/AR*P) and desensitized states. (**c**) Model 3: PTX binding prevents desensitization (that is, there is no ADP state).

To corroborate our desensitization gate model for inhibitory Cys-loop receptors, we used the pore blocker, picrotoxin (PTX), since its binding site is generally considered to be located deep in the pore (between −2′ and 2′) precisely where the pore constricts during the activation process (Fig. 5a)^14^,^23^. After applying a low GABA concentration (0.3 μM) to α1β2 receptors, enabling the resting (shut) state(s) of the receptor to remain significantly populated, we co-applied a saturating concentration (50 μM) of PTX, which blocked the GABA current. Subsequent washout of PTX while still in GABA revealed an extremely slow dissociation of the pore blocker (Fig. 5b), consistent with it being trapped in a resting (shut) state of the channel^23^. Repeating this protocol now with a saturating GABA concentration (10 mM) to deliberately drive receptors into the desensitized state, the apparent dissociation of PTX was much faster (Fig. 5c). This implies that PTX is not trapped in a desensitized state and that the desensitization gate must be distinct from the activation gate (Model 1, Fig. 6a).

Two simple schemes may explain the fast dissociation of PTX in the presence of saturating GABA. First, PTX binding/unbinding could be the same to open and desensitized receptor states. However, when GABA is saturating, the peak current after washing out PTX is clearly larger than the steady-state current before its application, which is not predicted by equal binding/unbinding to both states (Model 2, Fig. 6b). Second, PTX binding could be prevented in the desensitized state (Model 3, Fig. 6c). The recovery of current following PTX removal could then reflect two distinct processes: PTX dissociation from its binding site and subsequent receptor desensitization. For this scheme, we would expect the recovery current to exhibit a peak followed by a plateau. However, such behaviour is not obvious from our recordings with the GABA_A_R (Fig. 5c), probably because PTX dissociation is much slower than the rate of desensitization of GABA_A_Rs (Fig. 6c, Supplementary Table 2).

**Figure 7:**
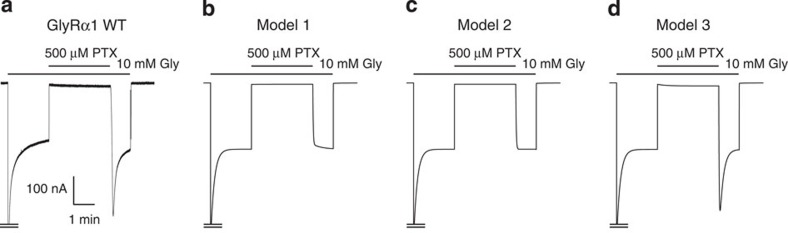
GlyR current recovery after blockade by PTX under desensitizing conditions. (**a**) Representative recording of the fast dissociation of PTX from GlyRα1 activated by high concentrations (10 mM) of glycine, accompanied by a clear rebound current (*n*=5). (**b**–**d**) Predicted membrane currents for activated wild-type GlyRα1 using the kinetic models 1–3, respectively, described in Fig. 6. See Supplementary Table 2 for the numerical values of parameters. Note, peak currents are truncated for clarity.

To gain a clearer view as to where PTX is binding in relation to our proposed desensitization locus, we examined GlyRs, where PTX dissociation is notably much faster^24^,^25^. During saturating glycine concentrations, PTX blocked the plateau current (Fig. 7a). Washing out PTX now revealed a clear peak-plateau current profile as expected theoretically, indicating that PTX had blocked the receptor in an open conformation, a profile that is accurately predicted only by Model 3 (Fig. 7b–d). These observations are consistent with a mechanism whereby the PTX-binding site spatially overlaps the desensitization gate. Furthermore, as desensitization leads to an increase in the agonist occupancy, such a model could account for the negative allosteric interaction between the receptor agonist and PTX^24^,^25^.

## Discussion

The present view of desensitization at Cys-loop receptors is that the molecular determinants are most likely contained within the ECD^26^ or the ECD–TMD coupling interface^27^,^28^. Our study indicates that a different domain is involved whereby the cytoplasmic end of the ion channel forms a physical desensitization gate in inhibitory Cys-loop receptors. While this may initially appear contradictory to earlier studies, a role for residues in the ECD or the ECD–TMD interface in setting macroscopic desensitization can be entirely reconciled with our new view. First, in the event that channel opening precedes desensitization, a mutation affecting the efficacy of gating could ultimately affect the macroscopic rate of desensitization without actually modifying the microscopic rate of desensitization. Interestingly, the ECD–TMD coupling interface is critical in setting the efficacy of channel gating. Accordingly, exchanging residues in this region between nAChRs and 5HT_3_Rs not only switches the desensitization properties of the receptors, but also their single-channel open lifetimes^27^.

Second, our proposal for a constriction (gate) of the channel lumen near its intracellular end during desensitization is likely to be accompanied by conformational rearrangements at the extracellular end that should be constrained by residues in the ECD–TMD coupling interface. Considering that M2 helices can exhibit rigid-body motion during gating^12^,^29^, we would predict that the pore may widen at its extracellular end during desensitization. This could be consistent with conformational rearrangements at the ECD–TMD interface of GABA_A_ receptors during desensitization, as reported by voltage-clamp fluorometry^28^. Moreover, such an effect has recently been proposed for GLIC, using electron paramagnetic resonance (EPR) spectroscopy to measure the distance between spin-labelled cysteines introduced along the ion channel pore^30^.

Interestingly, EPR predicts that desensitization might involve a mid-membrane gate with the intracellular end of the channel remaining mostly fixed. However, there are caveats to the interpretations of EPR data. First, in the narrowest part of the GLIC channel, spin labels might occlude the pore, thus acting as pore blockers, and as for PTX-bound GABA receptors, desensitization could conceivably be prevented by the labelling. Unfortunately, the irreversible inhibition by pore-blocking spin-labels would also render these receptors unsuitable for electrophysiological studies. Second, in the confined space of an ion channel pore, it is likely that the motion of the spin labels is also constrained. Thus, conformational changes around the spin labels could cause dynamic reorientation, making the extraction of distance information quite challenging^31^. For these reasons, EPR spectroscopy could be confounding when studying conformational rearrangements in the constricted space of an ion channel.

It is also important to note that a previous study showed that M1 and M2 were important in setting the desensitization properties of γ2 and δ subunit-containing GABA_A_ receptors^3^. While the molecular mechanisms were unclear, these results are interpretable in the light of our present study. The importance of M2 is clearly demonstrated by our mutagenesis data, and from our proposal regarding the location of a desensitization gate. Moreover, as there are extensive interactions between the TMDs, M1 would be expected to affect the conformation of both M2 and M3, and could also influence desensitization by affecting the M1–M2 linker. We should add a cautionary note to emphasize that perturbations to receptor structure can potentially have far reaching ramifications for predicting the consequences of mutagenesis to a functional outcome. For this reason, it is hard to predict the effects of residue substitution in the M2–M3 linker when manipulating the side-chain volume, charge and hydrophobicity. Nevertheless, structure–function studies of Cys-loop receptor activation, using similar methodologies to those employed here have proved to be very precise tools in identifying and attributing key parts of the receptor involved in function^12^,^29^, as evidenced by recent corroborating data between receptor function and the crystal structure of the apo form of GluCl^32^.

The molecular mechanisms underlying the desensitization of inhibitory Cys-loop receptors, which may conceivably extend to the entire Cys-loop family, appear strikingly different from the mechanisms responsible for desensitization of ionotropic GluRs. This is unsurprising, since from a structural point of view, Cys-loop receptors are quite distinct from ionotropic GluRs by forming pentamers with the ECD of each subunit interacting to form a ring, and the agonist binding sites located at interfaces between adjacent ECDs^7^,^33^. By contrast, ionotropic GluRs are tetramers harbouring discrete intrasubunit-binding sites^7^,^8^. Moreover, Cys-loop receptor subunits contain four transmembrane segments, which differ from the three transmembrane segments and one re-entrant P-loop topology of ionotropic GluR subunits.

The cytoplasmic end of the pore in Cys-loop receptors also contains the ion selectivity filter^34^. The conformational changes proposed to underlie desensitization here are reminiscent of the collapse of the selectivity filter that is considered responsible for slow inactivation of voltage-gated K^+^ and Na^+^ channels^35^,^36^,^37^. Specific mutations near the P-loop of these voltage-gated channels profoundly impact on slow inactivation, in a similar manner to the effects of the mutations described in this study for Cys-loop receptor desensitization^38^,^39^. Corroboration for the slow inactivation model of voltage-gated ion channels was obtained using pore-blockers, such as tetraethylammonium, which reduced slow inactivation of *Shaker* K^+^ channels by a ‘foot-in-the-door’ mechanism, essentially by binding near the selectivity filter to prevent its physical collapse^38^,^40^. By analogy, we used the Cys-loop Cl^−^ channel blocker PTX to prevent GABA_A_R and GlyR desensitization, which accorded with there being a spatial overlap between the desensitization gate and the PTX-binding site in the ion channel. Of note, a recent study showed that, in the absence of GABA, PTX is trapped in the resting state and not in the desensitized state^23^, though prolonged GABA applications were not investigated, and it therefore remains possible that PTX-bound receptors might desensitize in the presence of saturating concentrations of GABA. Indeed, all the models we depict (Fig. 6a–c) are consistent with the data in ref. 23. To extend this further, our experimental results show that PTX actually prevents desensitization even in the presence of saturating concentrations of GABA. Overall, our study identifies a new potential molecular mechanism in GABA_A_Rs and GlyRs that underpins one of the most fundamental parameters in pharmacology, that of receptor desensitization. We also establish a strong parallel between Cys-loop receptor desensitization and the slow inactivation of voltage-gated K^+^ and Na^+^ channels, even though the structure and membrane topology of these tetrameric channels is completely different from pentameric Cys-loop receptors.

In this context, it is notable that a recent crystal structure for the GABA receptor β3 homomer has been solved. Although it is considered not to be a physiological form of the GABA_A_ receptor, being unable to be activated by GABA^41^, the crystallographic data also alludes to a constriction at the intracellular end of the ion channel pore as being representative of a desensitized state^42^.

Our model suggests that the desensitization of Cys-loop receptors involves a rearrangement of the M2/M3 interface. This concept would accommodate the effects of modulators binding within the M1/M2/M3 intrasubunit cavity of nAChRs, which prevent desensitization^43^,^44^,^45^. Our results therefore provide a rationale for the mechanism of action of these drugs, and could help in designing pharmaceutical compounds to modulate Cys-loop receptor function. This is likely to be beneficial since Cys-loop receptors are involved in many pathological conditions, such as addiction, anxiety, depression, neurodegenerative diseases and seizures^46^,^47^,^48^.

## Methods

### Molecular biology

Murine GABA_A_R α1, β2 and γ2 subunits and human ρ1 subunits were subcloned into pRK5 at the EcoRI site. The human GlyRα1 subunit was subcloned into pRK5 between the EcoRI and the NotI sites. Most GABA_A_ chimeric receptors were obtained by amplification following a four-step procedure: first, a PCR was performed to build the amino-terminal end of the construct with the forward primer SP6 (5′ to 3′ sequence: CACATACGATTTAGGTGACACTATAG) and a reverse primer overlapping the junction between the parental DNAs, followed by DNA purification (QIAquick PCR purification kit, Qiagen). Second, a PCR was performed to build the C-terminal end of the construct with the reverse primer P5 (CAGACATGATAAGATACATTGATGAG) and a forward primer overlapping the junction between the parental DNAs, followed by DNA purification. Third, the PCR products were assembled through a final PCR step using SP6 and P5, followed by DNA gel extraction (QIAquick gel extraction kit, Qiagen). Fourth, the end product was subcloned into pRK5 with the restriction enzymes ClaI and SalI (NEB), using T4 DNA ligase (Roche). See Supplementary Table 4 for the primer sequences and the DNA templates used for the chimeras molecular biology. Single-point mutants and some chimeric receptors with small sequence exchanges (less than eight residues) were constructed with a single PCR reaction using Phusion DNA polymerase (Thermoscientific), followed by DNA gel extraction, 5′ phosphorylation using polynucleotide kinase (NEB) and subsequent ligation. See Supplementary Table 5 for the primer sequences and the DNA templates used for these mutant receptors.

### Two-electrode voltage clamp and analysis

*Xenopus laevis* oocytes expressing recombinant receptors were prepared, injected with complementary DNAs (cDNAs; at 1–30 ng μL^−1^), voltage clamped and recorded as described previously^16^. All compounds were purchased from Sigma. EDTA (10 μM) was added to the OR2 solution when recording from oocytes expressing GlyRs to chelate contaminating Zn^2+^, which potentiates GlyR function^49^. Recordings were digitized at 500 Hz and filtered at 50 Hz. Desensitizing currents were induced by 1 min agonist applications. The design of our recording chamber and the use of supersaturating concentrations of agonist enabled us to elicit currents with 20–80% rise times of 22 and 15 ms for wild-type α1β2 GABA_A_Rs and GlyRα1, respectively. The extent of desensitization was determined as




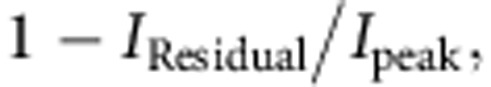




where *I*_peak_ is the agonist-induced peak current and *I*_Residual_ the residual of this current remaining at the end of the agonist application. Weighted decay time constants for desensitization were determined by fitting the desensitizing phase with two or three exponential components (Clampfit ver 8). Experiments that assessed the time period for recovery of the receptor from desensitization were performed by first driving the receptor into a profound desensitized state (30 s exposure to millimolar GABA or glycine concentrations), then monitoring the recovery of peak currents (2 s exposures) repeated at 10, 20, 30, 45, 60, 90, 120, 300 and 600-s intervals after the initial desensitizing exposure to the agonist.

To resolve even faster channel kinetics, we have performed rapid-application perfusion experiments with outside-out patches pulled from *Xenopus laevis* oocytes expressing GABA_A_Rs. Using a piezo-driven theta tube, we achieved 20–80% solution exchange times <100 μs. At wild-type α1β2 GABA_A_Rs, a 3-s exposure of 10 mM GABA led to a current that desensitized almost completely and is described by three exponential components of ∼10, 100 and 500 ms, consistent with published results obtained with recombinant receptors expressed in HEK cells^2^. Although the 10-ms component cannot be detected using two-electrode voltage clamp (TEVC), we should have resolved the 100-ms component using oocytes, given the 22-ms rise time for GABA-activated currents, and, because exponentials of this order were detected with some of our mutant receptors. However, we never resolved such a fast component at wild-type α1β2 GABA_A_Rs (fastest component was ∼1–2 s). Furthermore, the almost complete extent of fast desensitization in most outside-out patches did not concur with the large currents obtained with TEVC after a few seconds of exposure to GABA. Interestingly, recent results demonstrate that, in whole-cell patch-clamp recordings, GABA- and glycine-mediated currents decay much faster than their respective membrane conductances in the continuous presence of the agonist^50^. Since the decay rates of the currents were matched by the rates of change of internal Cl^−^ concentration, such an observation led the authors to speculate that the fast decay of currents, initially thought to reflect desensitization, actually reflects a decrease in the driving force for Cl^−^ ions. This effect is likely to be present in most patch-clamp experiments performed on small cells or outside-out patches, especially under high series resistance conditions. The oocyte is therefore a reliable model system to study the Cys-loop receptor desensitization. Of note, the slow conductance decay rates measured in ref. 50 are consistent with the desensitization kinetics we measure in TEVC at wild-type receptors.

Finally, as mentioned above, we used outside-out patches pulled from oocytes that expressed mutants showing increased rates of desensitization under TEVC. Unfortunately, we could not resolve any current from patches containing α1β2^ρ1^^(M1−M2 link)^ or α1^V251F^β2 receptors, even though the ‘parent’ oocytes exhibited strong functional expression (>5 μA peak GABA current). Moreover, using outside-out patches from oocytes expressing the mild mutant α1^G258A^β2^G254A^, we rarely recorded currents, and only then of a few pA decaying fully within 100 ms. For all these reasons, we discarded the fast-perfusion experiments.

### HEK whole-cell patch-clamp recording

HEK cells were transiently transfected using the calcium phosphate precipitation method with cDNAs pre-mixed in a 1:1 ratio, with 4 μg total cDNA applied to each per 22 mm coverslip. HEK cells were voltage clamped and recorded from as described previously^51^. Recordings were digitized and filtered at 6.5 kHz. Desensitizing currents were induced by 8 s applications of GABA (3 mM). The use of a rapid U-tube perfusion system and the use of supersaturating concentrations of agonist enabled us to elicit currents with average 20–80% rise times of <8 ms for wild-type α1β2 GABA_A_Rs. The extent of desensitization and weighted decay time constants were determined as for TEVC, up to 6 s after the start of GABA application.

### 3D molecular modelling and illustration

Modeller (ver 9.7 (ref. 52)) was used to build 3D homology models of the α1β2 heteromeric GABA_A_R based on the crystal structure of GluCl (pdb 3RHW (ref. 14)). Structures were visualized with Pymol^53^; pore radii were calculated with MOLE^54^.

### Kinetic modelling

Channelab (ver 2, Synaptosoft, GA) was used to build the virtual recordings in Figs 6 and 7. The binding and gating rate constants are broadly consistent with previously published values for GABA_A_Rs and GlyRs^51^,^55^. The desensitization rates were chosen to account for the profile of our current recordings. We modelled the slow component of receptor desensitization, since this is the component that is observed in our long duration applications of agonist, which were used to gather receptors in the desensitized state. The dissociation rate for PTX used for GABA_A_Rs predicted a slow recovery of the current consistent with our recordings in low concentrations of GABA. The dissociation constant for PTX (0.4 μM for GABA_A_Rs and 10 μM for GlyRs) are consistent with published IC_50_ values^24^,^56^.

## Additional information

**How to cite this article:** Gielen, M. *et al.* The desensitization gate of inhibitory Cys-loop receptors. *Nat. Commun.* 6:6829 doi: 10.1038/ncomms7829 (2015).

## Supplementary Material

Supplementary Information Supplementary Figures 1-9 and Supplementary Tables 1-5

## Figures and Tables

**Table 1:** Weighted decay time constant for desensitization (*τ*_W_) and extent of desensitization (% Des) of wild-type and mutant GABA_A_ and glycine receptor constructs.

**Construct**	***τ***_**W**_ **(s)**	**% Des**	***n***	**Construct**	***τ***_**W**_ **(s)**	**% Des**	***n***
α1β2 wt	17.4±3.6	86.4±2.6	17	α1^V251I^β2^S247I^	2.5±0.8	97.5±0.8	5
ρ1 wt	23.3±7.9	19±13	13	α1^V251D^β2^S247D^	1.3±0.4	93.5±1.2	4
				α1^V251F^β2	0.78±0.20	99.0±0.4	6
ρ1^Δ260^-α1	8.1±0.5	90.7±0.5	3	α1β2^S247F^	0.75±0.06	97.4±0.5	5
ρ1^Δ346^-α1	0.20±0.04	93.6±2.4	6	α1^N307Q^β2^N303Q^	11.4±1.0	90.8±0.4	4
ρ1^Δ433^-α1	28.2±2.6	11±4	3	α1^N307S^β2^N303S^	1.2±0.4	97±0.9	4
ρ1α^1(Δ6-postM3)^	1.0±0.4	72.2±4.2	9	α1^N307V^β2^N303V^	0.19±0.03	99.6±0.1	7
ρ1^T349K^	5.0±3.7	58.6±8.0	9	α1^N307D^β2	4.4±0.1	97.9±0.4	4
ρ1^β2(M1–M2 link)+^^α1(Δ6-postM3)^	10.5±3.3	25.3±8.6	10	α1β2^N303D^	0.41±0.12	87.1±4.1	4
α1^EXT^-ρ1^TM+INT^β2^EXT^-ρ1^TM+INT^	22.9±4.0	24.3±10.9	4	α1β2γ2L wt	15.5±3.2	79.8±4.5	8
α1β2^ρ1(M1–M2 link)^	0.33±0.11	98.9±0.2	11	α1β2γ2L^V262F^	1.3±0.2	98.7±0.2	7
α1^(Δ8-postM3)^β2^ρ1(Δ8-postM3)^	5.8±1.3	96.1±1.1	6	α1β2γ2L^H318V^	2.2±0.3	96.3±0.5	5
α1^(Δ8-postM3)^β2^ρ1(M1–M2 link+Δ8-postM3)^	4.6±1.5	80.4±6.2	7	GlyRα1 wt	10.7±3.4	72.4±8.1	12
α1^ρ1 M3^β2^ρ1 M3^	17.4±6.1	33.7±10.8	6	GlyRα1^ρ1(Δ6-postM3)^	0.88±0.23	98.2±1.0	6
α1^L300V^β2^L296V^	26.7±12.7	25.6±5.8	11	GlyRα1^L298V^	17.2±6.7	64.8±6.2	3
α1^G258A^β2^G254A^	4.9±1.4	93.5±1.5	12	GlyRα1^G256V^	0.84±0.16	99.6±0.3	9
α1^G258V^β2^G254V^	1.3±0.6	93.3±1.2	8	GlyRα1^V294L^	17.1±6.0	14.8±8.1	7
α1^V296L^β2^V292L^	93±15	30.4±3.3	5	GlyRα1^A249F^	0.85±0.65	98.0±0.9	4
α1^V251A^β2^S247A^	2.5±0.5	96.8±0.6	5	GlyRα1^N305V^	0.18±0.05	98.5±1.6	5

% Des, % desensitization; wt, wild type.

Values are means±s.d., *n* is the number of cells recorded for each construct.
