# Supplementary material for: The desensitization gate of inhibitory Cys-loop receptors
Source: Nat Commun. 2015 Apr 20;6:6829. doi: 10.1038/ncomms7829 (PMC4410641; doi:10.1038/ncomms7829)
Supplement: Supplementary Information — Supplementary Figures 1-9 and Supplementary Tables 1-5 [file ncomms7829-s1.pdf]

ρ1wt	251	YINFTLRRHIFFFLLQTYFPATLMVMLSWVSFWIDRRAVPARVPLGITTVLTMS	304
$\alpha_1$wt	213	TTHFHLKRKIGYFVIQTYLPCIMTVILSQVSFWLNRESVPARTVFGVTTVLTMT	266
$\beta_2$wt	209	SLSFKLKRNIGYFILQTYMPSILITILSWVSFWINYDASAARVALGITTVLTMT	262
GlyRα1wt	211	EARFHLERQMGYYLIQMYIPSLLIVILSWISFWINMDAAPARVGLGITTVLTMT	264
$\rho_1^{\Delta 260}$-α_1		YINFTLRRHIGYFVIQTYLPCIMTVILSQVSFWLNRESVPARTVFGVTTVLTMT	
α_1^{EXT}-ρ_1^{TM+INT}		TTHFHLKRKIFFFLLQTYFPATLMVMLSWVSFWIDRRAVPARVPLGITTVLTMS	
β_2^{EXT}-ρ_1^{TM+INT}		SLSFKLKRNIFFFLLQTYFPATLMVMLSWVSFWIDRRAVPARVPLGITTVLTMS	
$\rho_1^{\Delta 346}$-α_1		YINFTLRRHIFFFLLQTYFPATLMVMLSWVSFWIDRRAVPARVPLGITTVLTMS	
$\rho_1^{\Delta 433}$-α_1		YINFTLRRHIFFFLLQTYFPATLMVMLSWVSFWIDRRAVPARVPLGITTVLTMS	
$\rho_1^{\alpha 1(\Delta 6\text{-postM3})}$		YINFTLRRHIFFFLLQTYFPATLMVMLSWVSFWIDRRAVPARVPLGITTVLTMS	
$\rho_1^{\beta 2(\text{M1-M2 link})+\alpha 1(\Delta 6\text{-postM3})}$		YINFTLRRHIFFFLLQTYFPATLMVMLSWVSFWINYDASAARVPLGITTVLTMS	
$\beta_2^{\rho 1(\text{M1-M2 link})}$		SLSFKLKRNIGYFILQTYMPSILITILSWVSFWIDRRAVPARVALGITTVLTMT	
$\alpha_1^{\rho 1(\Delta 8\text{-postM3})}$		TTHFHLKRKIGYFVIQTYLPCIMTVILSQVSFWLNRESVPARTVFGVTTVLTMT	
$\beta_2^{\rho 1(\Delta 8\text{-postM3})}$		SLSFKLKRNIGYFILQTYMPSILITILSWVSFWINYDASAARVALGITTVLTMT	
$\beta_2^{\rho 1(\text{M1-M2 link + }\Delta 8\text{-postM3})}$		SLSFKLKRNIGYFILQTYMPSILITILSWVSFWIDRRAVPARVALGITTVLTMT	
GlyR$\alpha 1^{\rho 1(\Delta 6\text{-postM3})}$		EARFHLERQMGYYLIQMYIPSLLIVILSWISFWINMDAAPARVGLGITTVLTMT	
		-3′ 4′ 9′	

ρ1wt	305	TIITGVNASMPRVS-YIKAVDIYLWVSFVFVFLSVLEYAAVNYLTTVQERKEQ	356
$\alpha_1$wt	267	TLSISARNSLPKVA-YATAMDWFIAVCYAFVFSALIEFATVNYFTKRGYAWDG	318
$\beta_2$wt	263	TINTHLRETLPKIP-YVKAIDMYLMGCFVFVFMALLEYALVNYIFFGRGPQRQ	314
GlyRα1wt	265	TQSSGSRASLPKVS-YVKAIDIWMAVCLLFVFSALLEYAAVNFVSRQHKEQHK	316
$\rho_1^{\Delta 260}$-α_1		TLSISARNSLPKVA-YATAMDWFIAVCYAFVFSALIEFATVNYFTKRGYAWDG	
α_1^{EXT}-ρ_1^{TM+INT}		TIITGVNASLPKVA-YATAMDIYLWVSFVFVFLSVLEYAAVNYLTTVQERKEQ	
β_2^{EXT}-ρ_1^{TM+INT}		TIITGVNATLPKIP-YVKAIDIYLWVSFVFVFLSVLEYAAVNYLTTVQERKEQ	
$\rho_1^{\Delta 346}$-α_1		TIITGVNASMPRVS-YIKAVDIYLWVSFVFVFLSVLEYAAVNYFTKRGYAWDG	
$\rho_1^{\Delta 433}$-α_1		TIITGVNASMPRVS-YIKAVDIYLWVSFVFVFLSVLEYAAVNYLTTVQERKEQ	
$\rho_1^{\alpha 1(\Delta 6\text{-postM3})}$		TIITGVNASMPRVS-YIKAVDIYLWVSFVFVFLSVLEYAAVNYFTKRGYRKEQ	
$\rho_1^{\beta 2(\text{M1-M2 link})+\alpha 1(\Delta 6\text{-postM3})}$		TIITGVNASMPRVS-YIKAVDIYLWVSFVFVFLSVLEYAAVNYFTKRGYRKEQ	
$\beta_2^{\rho 1(\text{M1-M2 link})}$		TINTHLRETLPKIP-YVKAIDMYLMGCFVFVFMALLEYALVNYIFFGRGPQRQ	
$\alpha_1^{\rho 1(\Delta 8\text{-postM3})}$		TLSISARNSLPKVA-YATAMDWFIAVCYAFVFSALIEFATVNYLTTVQERKDG	
$\beta_2^{\rho 1(\Delta 8\text{-postM3})}$		TINTHLRETLPKIP-YVKAIDMYLMGCFVFVFMALLEYALVNYLTTVQERKRQ	
$\beta_2^{\rho 1(\text{M1-M2 link + }\Delta 8\text{-postM3})}$		TINTHLRETLPKIP-YVKAIDMYLMGCFVFVFMALLEYALVNYLTTVQERKRQ	
GlyR$\alpha 1^{\rho 1(\Delta 6\text{-postM3})}$		TQSSGSRASLPKVS-YVKAIDIWMAVCLLFVFSALLEYAAVNFVSTVQERKHK	

ρ1wt	429	IDTHAIDKYSRIIFPAAYILFNLIYWSIFS------	458
$\alpha_1$wt	386	NSVSKIDRLSRIAFPLLFGIFNLVYWATYLNREPQL	421
$\beta_2$wt	420	TDVNAIDRWSRIFFPVVFSFFNIVYWLYYVN-----	450
GlyRα1wt	382	QRAKKIDKISRIGFPMAFLIFNMFYWIIYKIVRRED	417
$\rho_1^{\Delta 260}$-α_1		NSVSKIDRLSRIAFPLLFGIFNLVYWATYLNREPQL	
α_1^{EXT}-ρ_1^{TM+INT}		IDTHAIDKYSRIIFPAAYILFNLIYWSIFS------	
β_2^{EXT}-ρ_1^{TM+INT}		IDTHAIDKYSRIIFPAAYILFNLIYWSIFS------	
$\rho_1^{\Delta 346}$-α_1		NSVSKIDRLSRIAFPLLFGIFNLVYWATYLNREPQL	
$\rho_1^{\Delta 433}$-α_1		IDTHAIDRLSRIAFPLLFGIFNLVYWATYLNREPQL	
$\rho_1^{\alpha 1(\Delta 6\text{-postM3})}$		IDTHAIDKYSRIIFPAAYILFNLIYWSIFS------	
$\rho_1^{\beta 2(\text{M1-M2 link})+\alpha 1(\Delta 6\text{-postM3})}$		IDTHAIDKYSRIIFPAAYILFNLIYWSIFS------	
$\beta_2^{\rho 1(\text{M1-M2 link})}$		TDVNAIDRWSRIFFPVVFSFFNIVYWLYYVN-----	
$\alpha_1^{\rho 1(\Delta 8\text{-postM3})}$		NSVSKIDRLSRIAFPLLFGIFNLVYWATYLNREPQL	
$\beta_2^{\rho 1(\Delta 8\text{-postM3})}$		TDVNAIDRWSRIFFPVVFSFFNIVYWLYYVN-----	
$\beta_2^{\rho 1(\text{M1-M2 link + }\Delta 8\text{-postM3})}$		TDVNAIDRWSRIFFPVVFSFFNIVYWLYYVN-----	
GlyR$\alpha 1^{\rho 1(\Delta 6\text{-postM3})}$		QRAKKIDKISRIGFPMAFLIFNMFYWIIYKIVRRED	

Supplementary Figure 1. Alignment of wild-type and chimeric receptor subunits.

The transmembrane segments are shaded. Singly mutated residues are highlighted in yellow. The sequences are colour-coded, black, green, red and blue for residues found in ρ1, α1, β2 and GlyRα1, respectively. Most of the ECD and the M3-M4 loop are omitted.

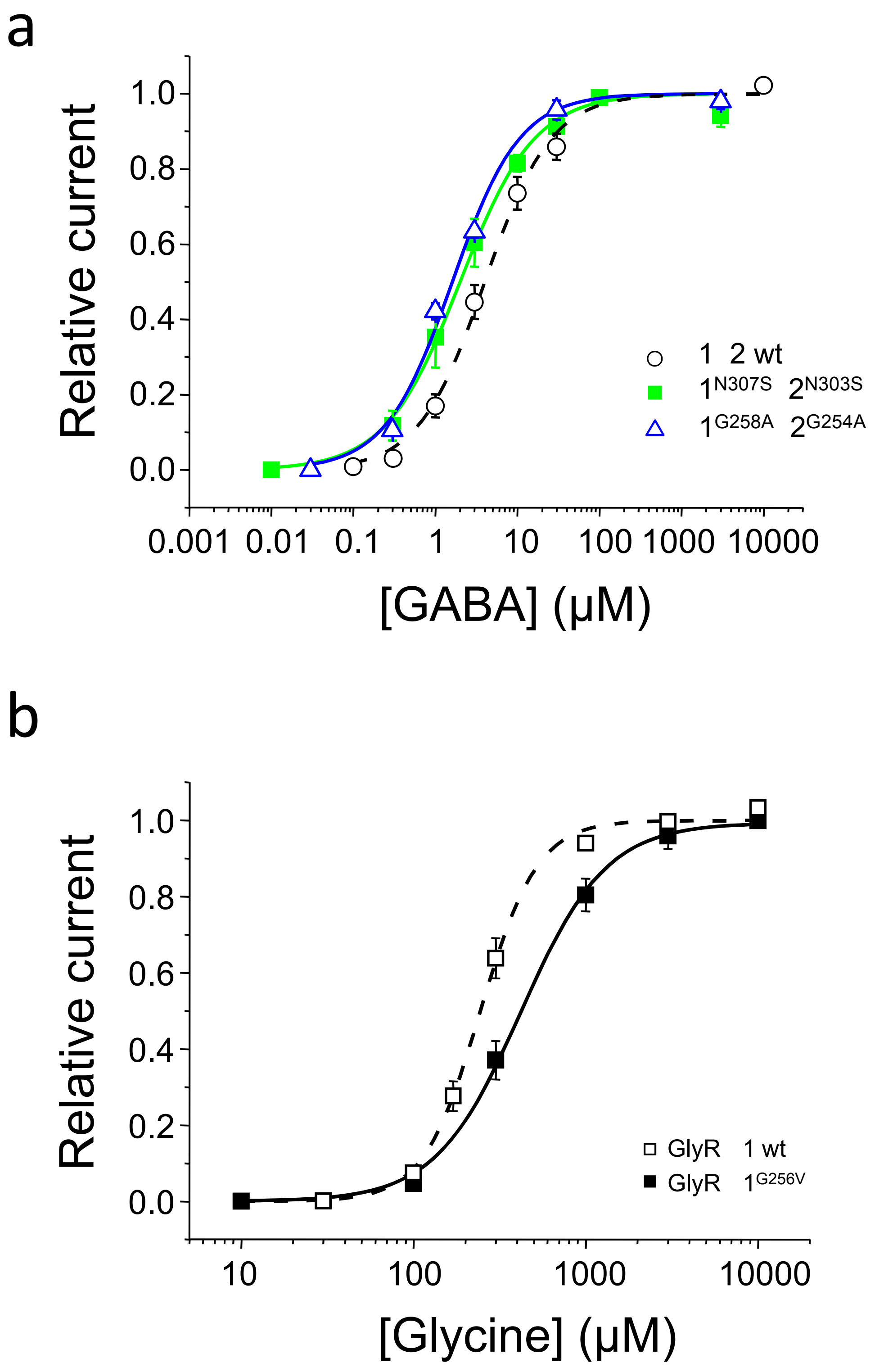
a
Relative current
1.0
0.8
0.6
0.4
0.2
0.0
0.001 0.01 0.1 1 10 100 1000 10000
[GABA] (µM)
1 2 wt
1N307S 2N303S
1G258A 2G254A
b
Relative current
1.0
0.8
0.6
0.4
0.2
0.0
10 100 1000 10000
[Glycine] (µM)
GlyR 1 wt
GlyR 1G256V

Supplementary Figure 2. Effects of mutations on GABA and glycine sensitivity.

(a) GABA concentration-response curves for $\alpha 1\beta 2$ wild-type (wt) (EC_{50} = 4.1 ± 0.7 µM, n_H = 1.08 ± 0.07, n = 6), and the mutant receptors: $\alpha 1^{N307S}\beta 2^{N303S}$ (EC_{50} = 2.0 ± 1.1 µM, n_H = 1.16 ± 0.13, n = 5) and $\alpha 1^{G258A}\beta 2^{G254A}$ (EC_{50} = 1.7 ± 0.4 µM, n_H = 0.87 ± 0.16, n = 6). All points are mean ± s.d.

(b) Glycine concentration-response curve for wild-type homomeric GlyR α1 wild type (wt) (EC_{50} = 245 ± 19 µM, n_H = 2.65 ± 0.07, n = 3) and mutant $\alpha 1^{G256V}$ (EC_{50} = 424 ± 56 µM, n_H = 1.77 ± 0.11, n = 3).

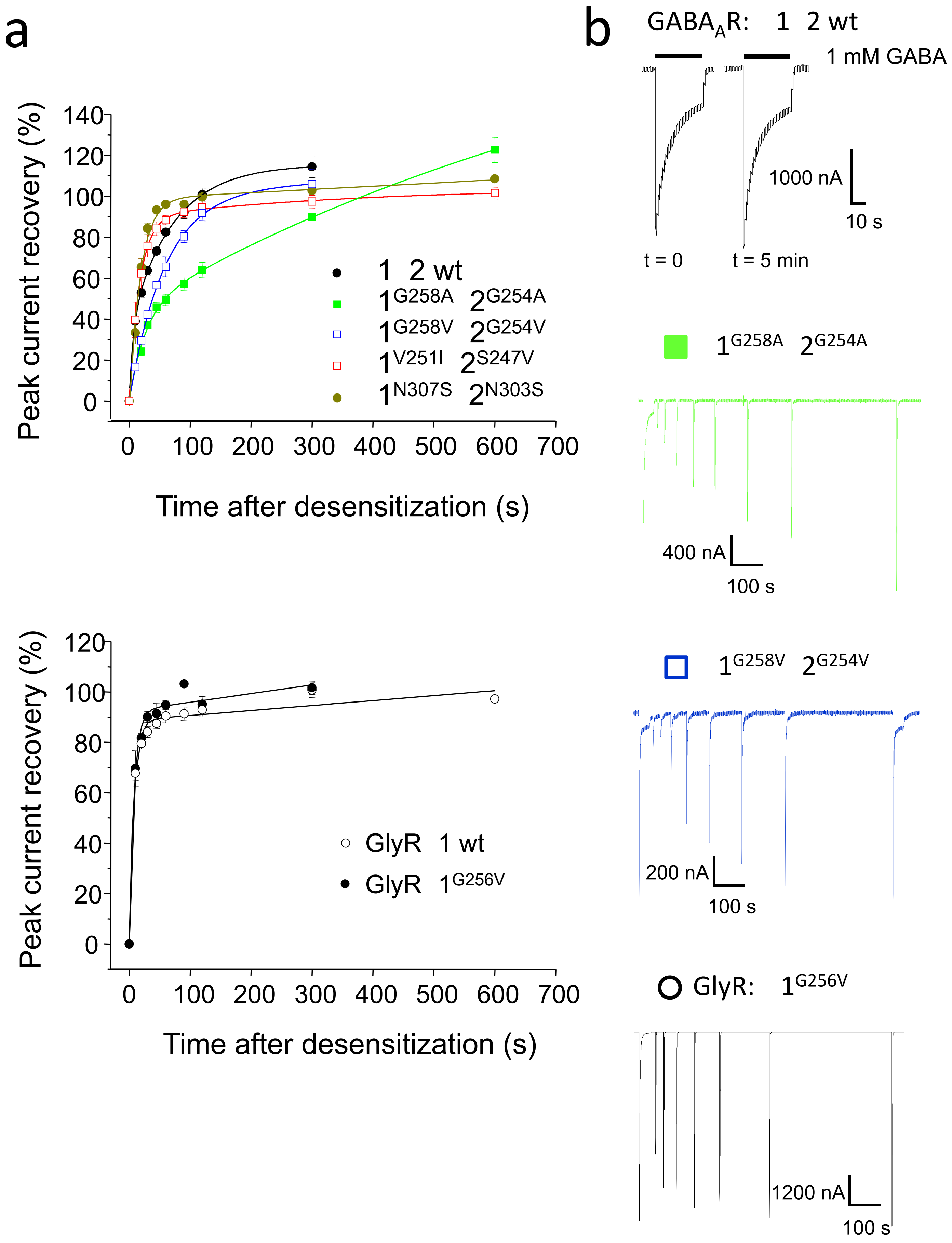

a
b
Peak current recovery (%)
Time after desensitization (s)
1 2 wt
1G258A 2G254A
1G258V 2G254V
1V251I 2S247V
1N307S 2N303S
GlyR 1 wt
GlyR 1G256V
GABAAR: 1 2 wt
1 mM GABA
1000 nA
10 s
t = 0
t = 5 min
400 nA
100 s
200 nA
100 s
GlyR: 1G256V
1200 nA
100 s

Supplementary Figure 3. Recovery from desensitization.

(a) Time-course of peak current recovery for the wild type (wt) and mutant $GABA_AR$ and GlyR constructs indicated, expressed in oocytes, following an initial, strongly desensitizing (30 s) challenge with either GABA (1 mM) or glycine (3 mM). Receptors were subsequently exposed to the same agonist concentration (for 2 s) at 10, 20, 30, 45, 60, 90, 120, 300 and 600 s intervals after the initial agonist exposure to monitor the recovery phase. The peak current of the first challenge is defined as 100 %. The lines are non-linear least squares fits using a double exponential function.

(b) Top panel: response profiles for the $\alpha1\beta2$ wild-type $GABA_AR$ following an initial desensitizing GABA challenge (1 mM for 30 s, black bar, t = 0) and that showing complete recovery (after 300 s / 5 min). Lower panels depict representative peak currents for the receptor constructs indicated, showing the initial agonist challenge (30 s) to induce desensitisation and the subsequent current recovery (Note: 600 s time point is excluded for clarity).

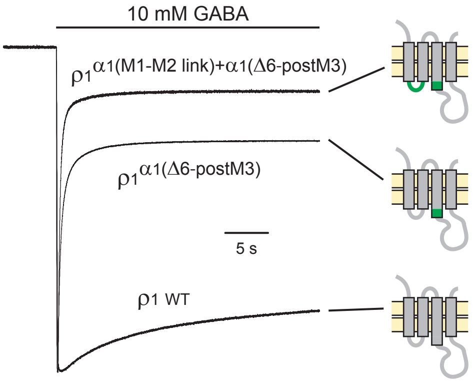

Supplementary Figure 4. Desensitisation of $\rho 1^{\alpha 1(M1\text{-}M2\ link)+\alpha 1(\Delta 6\text{-}postM3)}$ receptors.

Left: recordings of currents elicited by 10 mM GABA, showing the desensitisation of the indicated receptors. Note that the incorporation of the α1 M1-M2 linker did not reverse the gain of function obtained after incorporation of the C-terminal end of the α1 M3 segment. Similar results were obtained for 5 oocytes expressing $\rho 1^{\alpha 1(M1\text{-}M2\ link)+\alpha 1(\Delta 6\text{-}postM3)}$ receptors. Right: the chimeras are depicted by a colour code, green for α1 and grey for ρ1.

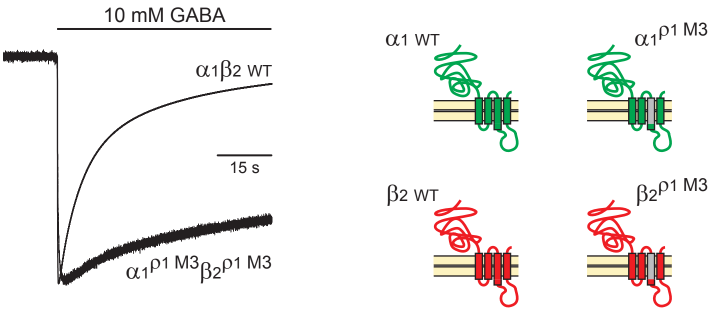

Supplementary Figure 5. Desensitisation of $\alpha1^{\rho1\ M3}\beta2^{\rho1\ M3}$ receptors.

Left: recordings of currents elicited by 10 mM GABA, showing the desensitisation of the indicated receptors. Similar results were obtained for 6 oocytes expressing $\alpha1^{\rho1\ M3}\beta2^{\rho1\ M3}$ receptors. Right: the chimeras are depicted by a colour code, green for α1, red for β2 and grey for ρ1.

		* ** **	
GABA$_A$R ρ1	251	YINFTLRRHIFFFLLQTYFPATLMVMLSWVSFWIDRRAVPARVPLGITTVLTMS	304
ρ2		YINFTLRRHIFFFLLQTYFPATLMVMLSWVSFWIDHRAVPARVSLGIMTVLTMS	
ρ3		FINFVLRRHIFFFVLQTYFPAMLMVMLSWVSFWIDRRAVPARVSLGITTVLTMS	
α1	213	TTHFHLKRKIGYFVIQTYLPCIMTVILSQVSFWLNRESVPARTVFGVTTVLTMT	266
α2		TAHFHLKRKIGYFVIQTYLPCIMTVILSQVSFWLNRESVPARTVFGVTTVLTMT	
α3		TTHFHLKRKIGYFVIQTYLPCIMTVILSQVSFWLNRESVPARTVFGVTTVLTMT	
α5		TAHFHLKRKIGYFVIQTYLPCIMTVILSQVSFWLNRESVPARTVFGVTTVLTMT	
α4		TVYFHLRRKMGYFMIQTYIPCIMTVILSQVSFWINKESVPARTVFGITTVLTMT	
α6		TVYFHLQRKMGYFMIQIYTPCIMTVILSQVSFWINKESVPARTVFGITTVLTMT	
β1		SLSFRLKRNIGYFILQTYMPSTLITILSWVSFWINYDASAARVALGITTVLTMT	
β2	209	SLSFKLKRNIGYFILQTYMPSILITILSWVSFWINYDASAARVALGITTVLTMT	262
β3		SLSFRLKRNIGYFILQTYMPSILITILSWVSFWINYDASAARVALGITTVLTMT	
γ 1		TIFFDLSRRMGYFTIQTYIPCILTVVLSWVSFWINKDAVPARTSLGITTVLTMT	
γ 2		SVYFDLSRRMGYFTIQTYIPCTLIVVLSWVSFWINKDAVPARTSLGITTVLTMT	
γ 3		TIYFELSRRMGYFTIQTYIPCILTVVLSWVSFWIKKDATPARTTLGITTVLTMT	
δ		SLHFQLRRNRGVYIIQSYMPSVLLVAMSWVSFWISQAAVPARVSLGITTVLTMT	
GlyR α1	211	EARFHLERQMGYYLIQMYIPSLLIVILSWISFWINMDAAPARVGLGITTVLTMT	264
α2		EVKFHLERQMGYYLIQMYIPSLLIVILSWVSFWINMDAAPARVALGITTVLTMT	
α3		EVRFHLERQMGYYLIQMYIPSLLIVILSWVSFWINMDAAPARVALGITTVLTMT	
α4		EVKFHLERQMGYYLIQMYIPSLLIVILSWVSFWINMDAAPARVGLGITTVLTMT	
β		EVIFTLRRQVGFYMMGVYAPTLLIVVLSWLSFWINPDASAARVPLGIFSVLSLA	
GluCl α1		RTTIQLKREFSFYLLQLYIPSCMLVIVSWVSFWFDRTAIPARVTLGVTTLLTMT	
GluCl β		RMQLLFKRQFSYYLVQLYAPTTMIVIVSWVSFWIDLHSTAGRVALGVTTLLTMT	
nAChR α7		TYTVTMRRRTLYYGLNLLIPCVLISALALLVFLLPADSG-EKISLGITVLLSLT	
5HT3A		KFYVIIRRRPLFYAVSLLLPSIFLMVVDIVGFCLPPDSG-ERVSFKITLLLGYS	
GLIC		DYQLRISRQYFSYIPNIILPMLFILFISWTAFWS--TSYEANVTLVVSTLIAHI	
ELIC		TVRIDAVRNPSYYLWSFILPLGLIIAASWSVFWL--ESFSERLQTSFTLMLTVV	

M1 M2

		** ** *	
GABA$_A$R ρ1	305	TIITGVNASMPRVS-YIKAVDIYLWVSFVFVFLSVLEYAAVNYLTTVQERKEQ	356
ρ2		TIITGVNASMPRVS-YIRAVDIYLWVSFVFVFLSVLEYAAVNYLTTLQEQKEL	
ρ3		TIVTGVSASMPQVS-YVKAVDVYMWVSSLFVFLSVIEYAAVNYLTTVEEWKQL	
α1	267	TLSISARNSLPKVA-YATAMDWFIAVCYAFVFSALIEFATVNYFTKRGYAWDG	318
α2		TLSISARNSLPKVA-YATAMDWFIAVCYAFVFSALIEFATVNYFTKRGWAWDG	
α3		TLSISARNSLPKVA-YATAMDWFIAVCYAFVFSALIEFATVNYFTKRSWAWEG	
α5		TLSISARNSLPKVA-YATAMDWFIAVCYAFVFSALIEFATVNYFTKRGWAWDG	
α4		TLSISARHSLPKVS-YATAMDWFIAVCFAFVFSALIEFAAVNYFTNIQMQKAK	
α6		TLSISARHSLPKVS-YATAMDWFIAVCFAFVFSALIEFAAVNYFTNLQSQKAE	
β1		TISTHLRETLPKIP-YVKAIDIYLMGCFVFVFLALLEYAFVNYIFFGKGPQKK	
β2	263	TINTHLRETLPKIP-YVKAIDMYLMGCFVFVFMALLEYALVNYIFFGRGPQRQ	314
β3		TINTHLRETLPKIP-YVKAIDMYLMGCFVFVFLALLEYAFVNYIFFGRGPKKL	
γ 1		TLSTIARKSLPKVS-YVTAMDLFVSVCFIFVFAALMEYGTLHYFTSNNKGKTT	
γ 2		TLSTIARKSLPKVS-YVTAMDLFVSVCFIFVFSALVEYGTLHYFVSNRKPSKD	
γ 3		TLSTIARKSLPRVS-YVTAMDLFVTVCFLFVFAALMEYATLNYYSSCRKPTIR	
δ		TLMVSARSSLPRAS-AIKALDVYFWICYVFVFAALVEYAFAHFNADYRKKRKA	
GlyR α1	265	TQSSGSRASLPKVS-YVKAIDIWMAVCLLFVFSALLEYAAVNFVSRQHKELLR	316
α2		TQSSGSRASLPKVS-YVKAIDIWMAVCLLFVFAALLEYAAVNFVSRQHKELLR	
α3		TQSSGSRASLPKVS-YVKAIDIWMAVCLLFVFSALLEYAAVNFVSRQHKEFLR	
α4		TQSSGSRASLPKVS-YVKAIDIWMAVCLLFVFAALLEYAAVNFVSRQHKEFMR	
β		SECTTLAAELPKVS-YVKALDVWLIACLLFGFASLVEYAVVQVMLNNPKRVEA	
GluCl α1		AQSAGINSQLPPVS-YIKAIDVWIGACMTFIFCALLEFALVNHIANKQGVERQ	
GluCl β		TMQSAINAKLPPVS-YVKVVDVWLGACQTFVFGALLEYAFVSYQDSVRQNDRR	
nAChR α7		VFMLLVAEIMPATSDSVPLIAQYFASTMIIVGLSVVVTVIVLRYHHHDPDGGK	
5HT3A		VFLIIVSDTLPATIG-TPLIGVYFVVCMALLVISLAETIFIVRLVHKQDLQRP	
GLIC		AFNILVETNLPKTP-YMTYTGAIIFMIYLFYFVAVIEVTVQHYLKVESQPAR-	
ELIC		AYAFYTSNILPRLP-YTTVIDQMIIAGYGSIFAAILLIIFAHHRQAMGVE---	

M2 M3

GABA$_A$R ρ1	429	IDTHAIDKYSRIIFPAAYILFNLIYWSIFS------	458
ρ2		QNTHAIDKYSRLIFPAFYIVFNLIYWSVFS------	
ρ3		ENNHVIDTYSRIVFPVVYIIFNLFYWGIYV------	
α1	386	NSVSKIDRLSRIAFPLLFGIFNLVYWATYLNREPQL	421
α2		NSVSKIDRMSRIVFPVLFGTFNLVYWATYLNREPVL	
α3		NSVSKVDKISRIIFPVLFAIFNLVYWATYVNRESAI	
α5		NSISKIDKMSRIVFPILFGTFNLVYWATYLNREPVI	
α4		SGTSKIDKYARILFPVTFGAFNMVYWVVYLSKDTME	
α6		GGTSKIDQYSRILFPVAFAGFNLVYWIVYLSKDTME	
β1		TDVNSIDKWSRMFFPITFSLFNVVYWLYYVH-----	
β2	420	TDVNAIDRWSRIFFPVVFSFFNIVYWLYYVN-----	450
β3		TDVNAIDRWSRIVFPFTFSLFNLVYWLYYVN-----	
γ1		IRIAKIDSYSRIFFPTAFALFNLVYWVGYLYL----	
γ2		IRIAKMDSYARIFFPTAFCLFNLVYWVSYLYL----	
γ3		IDVSELDSYSRVFFPTSFLLFNLVYWVGYLYL----	
δ		IDADTIDIYARAVFPAAFAAVNIIYWAAYTM-----	
GlyR α1	382	QRAKKIDKISRIGFPMAFLIFNMFYWIIYKIVRRED	417
α2		DRAKRIDTISRAAFPLAFLIFNIFYWITYKIIRHED	
α3		DRAKKIDTISRACFPLAFLIFNIFYWVIYKILRHED	
α4		DRAKRIDTISRAVFPFTFLVFNIFYWVVYKVLRSED	
β		TAAKRIDLYARALFPFCFLFFNVIYWSIYL------	
GluCl α1		DISKRVDLISRALFPVLFFVFNILYWSRFGQQNVLF	
GluCl β		YLPAKIDFYARFVVPLAFLAFNVIYWVSCLIMSANA	
nAChR α7		FAACVVDRLCLMAFSVFTIICTIGILMSAPNFVEAV	
5HT3A		RVGYVLDRLLFRIYLLAVLAYSITLVTLWSIWHYS-	
GLIC		--AASITRASRIAFPVVFLLANIILAFLFFGF----	
ELIC		--DDLLIQRCRLAFPLGFLAIGCVLVIRGITL----	
		M4	

Supplementary Figure 6. Alignment of the transmembrane segments of various members of the Cys-loop family.

The extracellular domain (~ 200 first residues) and the M3-M4 intracellular loop are not shown. All sequences are the mouse orthologs, except ρ1 (human), GluCl (*Caenorhabditis elegans*), GLIC (*Gloeobacter violaceus*) and ELIC (*Erwinia chrysanthemi*). The transmembrane segments are shaded in grey and depicted below the sequences. The intracellular end of M3 is highlighted in dark salmon. The numbering of residues is shown for subunits used in the present work. Singly mutated residues in this study are highlighted in yellow. Pore lining residues in M2 are shown by red stars according to the GABA β subunit.

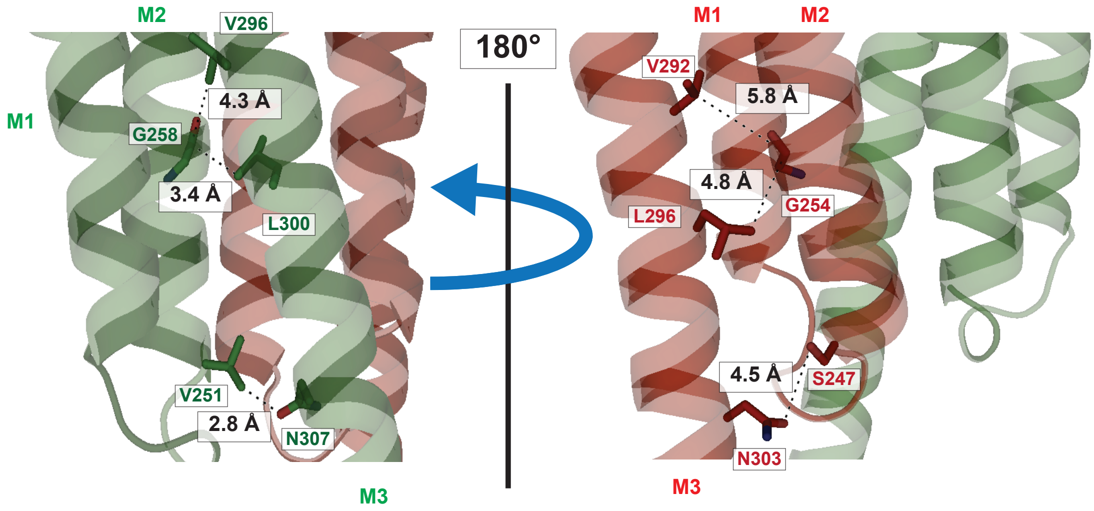

Supplementary Figure 7. Distance measurements between key M2 and M3 residues

Distances are indicated for key α1 residues in green, and key β2 residues in red. Note, compared to Fig. 2b, the viewing angle of the subunit interface is rotated by 180°.

a

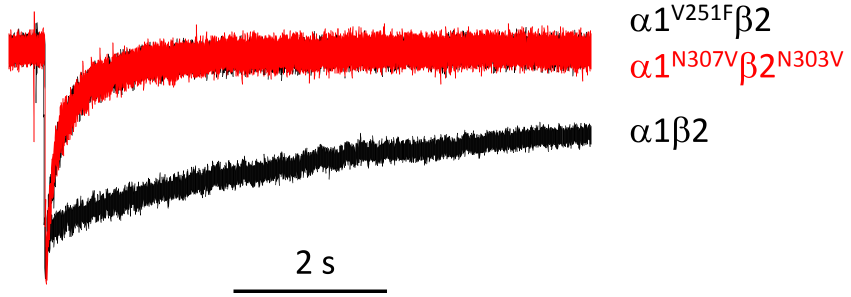

b

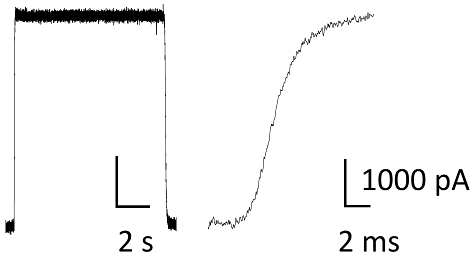

Supplementary Figure 8. Desensitisation of wild-type and mutant GABA$_A$Rs expressed in HEK cells.

Heteromeric α1β2 GABA$_A$ receptors were expressed in HEK cells. (**a**) Using rapid application of 3 mM GABA, recordings of GABA membrane currents were obtained showing differential levels of desensitisation relative to wild-type GABA$_A$ receptors. Note the GABA current for α1^{V251F}β2 (black trace) is overlaid by that for α1^{N307V}β2^{N303V} (red trace). (**b**) Depicts a typical patch electrode open tip junction potential change in response to a change in solution tonicity (Krebs to 50% v/v Krebs). The entirety of the response is shown (left) together with the 20 – 80% response time, typically 3 ms. For all HEK cells used in this study, the saturating GABA responses all had rise times < 5 - 10 ms.

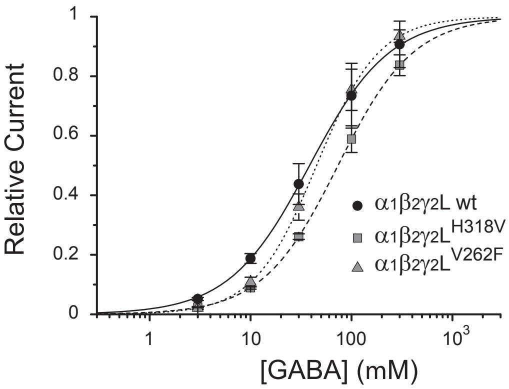

Supplementary Figure 9. Effects of mutations α1β2γ2L^{H318V} and α1β2γ2L^{V262F} on GABA sensitivity.

GABA dose-response curve of α1β2γ2L wt (EC_{50} = 43 ± 17 μM, nH = 1.09 ± 0.20 [n = 3]), α1β2γ2L^{H318V} (EC_{50} = 75 ± 8 μM, nH = 1.16 ± 0.11 [n = 4]) and α1β2γ2L^{V262F} receptors (EC_{50} = 46 ± 7 μM, nH = 1.41 ± 0.13 [n = 4]). Error bars represent s.d.

Construct	Peak current	n
$\alpha1\beta2$ wt	1742 ± 1363	18
$\rho1$ wt	576 ± 525	14
$\rho1^{\Delta260}$-$\alpha1$	1849 ± 604	3
$\rho1^{\Delta346}$-$\alpha1$	119 ± 114	7
$\rho1^{\Delta433}$-$\alpha1$	145 ± 67	3
$\rho1^{\alpha1(\Delta6\text{-postM3})}$	1068 ± 647	9
$\rho1^{T349K}$	1318 ± 1826	10
$\rho1^{\beta2(\text{M1-M2 link})+\alpha1(\Delta6\text{-postM3})}$	614 ± 546	10
$\alpha1^{EXT}$-$\rho1^{TM+INT}$<br>$\beta2^{EXT}$-$\rho1^{TM+INT}$	280 ± 329	8
$\alpha1\beta2^{\rho1(\text{M1-M2 link})}$	1980 ± 1778	11
$\alpha_1{}^{\rho1(\Delta8\text{-postM3})}\beta_2{}^{\rho1(\Delta8\text{-postM3})}$	2823 ± 1606	6
$\alpha_1{}^{\rho1(\Delta8\text{-postM3})}$<br>$\beta_2{}^{\rho1(\text{M1-M2 link} + \Delta8\text{-postM3})}$	1492 ± 939	7
$\alpha_1{}^{\rho1\ M3}\beta_2{}^{\rho1\ M3}$	47 ± 29	6
$\alpha1^{L300V}\beta2^{L296V}$	185 ± 122	13
$\alpha1^{G258A}\beta2^{G254A}$	397 ± 561	12
$\alpha1^{G258V}\beta2^{G254V}$	286 ± 194	7
$\alpha1^{V296L}\beta2^{V292L}$	177 ± 162	5
$\alpha1^{V251A}\beta2^{S247A}$	1880 ± 1394	5

Construct	Peak current	n
$\alpha1^{V251I}\beta2^{S247I}$	1126 ± 1211	5
$\alpha1^{V251D}\beta2^{S247D}$	74 ± 35	4
$\alpha1^{V251F}\beta2$	3336 ± 1161	6
$\alpha1\beta2^{S247F}$	1652 ± 779	5
$\alpha1^{N307Q}\beta2^{N303Q}$	2509 ± 981	4
$\alpha1^{N307S}\beta2^{N303S}$	3655 ± 3549	5
$\alpha1^{N307V}\beta2^{N303V}$	2416 ± 1364	7
$\alpha1^{N307D}\beta2$	2283 ± 1127	5
$\alpha1\beta2^{N303D}$	202 ± 90	4
$\alpha1\beta2\gamma2L$ wt	2956 ± 2057	8
$\alpha1\beta2\gamma2L^{V262F}$	1616 ± 1052	7
$\alpha1\beta2\gamma2L^{H318V}$	915 ± 871	5
GlyR$\alpha1$ wt	1657 ± 1320	12
GlyR$\alpha1^{\rho1(\Delta6\text{-postM3})}$	1568 ± 2011	7
GlyR$\alpha1^{L298V}$	3703 ± 689	3
GlyR$\alpha1^{G256V}$	1604 ± 1129	9
GlyR$\alpha1^{V294L}$	233 ± 481	7
GlyR$\alpha1^{A249F}$	774 ± 845	4
GlyR$\alpha1^{N305V}$	786 ± 873	6

Supplementary Table 1. Average whole-cell peak current sizes (nA) of wild-type and mutant $GABA_A$ and glycine receptor constructs. Drug concentration was 10 mM GABA or glycine, except GlyR$\alpha1^{L298V}$ (100 mM glycine). Values are means ± s.d., n is the number of cells recorded for each construct.

Rate	$\alpha 1\beta 2$ WT	GlyRα1 WT
k_1 ($M^{-1}.s^{-1}$)	10^7	$7.5x10^4$
k_{-1} (s^{-1})	300	300
α (s^{-1})	350	$5x10^3$
β (s^{-1})	$2.5x10^3$	10^5
δ^+ (s^{-1})	$3.3x10^{-2}$	$6x10^{-2}$
δ^- (s^{-1})	$6.7x10^{-3}$	$4x10^{-2}$
k_2 ($M^{-1}.s^{-1}$)	$7x10^4$	$8x10^4$
k_{-2} (s^{-1})	$3x10^{-2}$	0.8

Supplementary Table 2. Parameters used to generate the kinetic models in Fig. 6 (see Methods).

Construct	Amplitude (pA)	τ_W (ms)	% Des	n
α1β2	1267.2 ± 258	2675 ± 379	66.4 ± 2.8	13-17
$\alpha1^{V251F}\beta2$	248.1 ± 40	280 ± 57	100	13-14
$\alpha1^{N307V}\beta2^{N303V}$	104.8 ± 30.5	410.1 ± 77	100	6-9

Supplementary Table 3. GABA (3 mM) -activated current amplitudes, weighted decay time constants (τ_w) for desensitisation and the extents of desensitization (% Des) are shown for key selected $GABA_AR$ constructs expressed in HEK cells. Values represent mean ± s.e.m., and n is the number of cells recorded for each construct.

Construct	Primers & template	PCR 1 (N-terminal)	PCR 2 (C-Terminal)	PCR 3 (assembly)
$\rho1^{\Delta260}$-$\alpha1$	Forward	SP6	CGTCGCCACATCGGCTACTTTGTTATTCAAACATA	SP6
	Reverse	AACAAAGTAGCCGATGTGGCGACGCAACGTG	P5	P5
	Template	ρ1 wt	α1 wt	PCR 1 + 2
$\rho1^{\Delta346}$-$\alpha1$	Forward	SP6	GGCCGTCAACTACTTCACCAAGAGAGGGTATGCG	SP6
	Reverse	CTCTCTTGGTGAAGTAGTTGACGGCCGCATACTC	P5	P5
	Template	ρ1 wt	α1 wt	PCR 1 + 2
$\rho1^{\Delta433}$-$\alpha1$	Forward	SP6	ACCCACGCCATCGACCGACTGTCAAGAATAG	SP6
	Reverse	CAGTCGGTCGATGGCGTGGGTGTCGATTC	P5	P5
	Template	ρ1 wt	α1 wt	PCR 1 + 2
$\alpha1^{\Delta222}$-$\rho1$	Forward	SP6	AAGAGAAAAATTTTCTTCTTCTTGCTCCAAAC	SP6
	Reverse	CAAGAAGAAGAAAATTTTTCTCTTCAAGTGGAAG	P5	P5
	Template	α1 wt	ρ1 wt	PCR 1 + 2
$\alpha1^{\rho1(M1+M2)}$	Forward	SP6	CGTGAATGCCTCCCTCCCGAAGGTGG	SP6
	Reverse	CTTCGGGAGGGAGGCATTCACGCCCGTG	P5	P5
	Template	$\alpha1^{\Delta222}$-$\rho1$	α1 wt	PCR 1 + 2
$\alpha1^{EXT}$-$\rho1^{TM+INT}$	Forward	SP6	CTTATGCAACAGCTATGGACATCTACCTCTGGGTCAGC	SP6
	Reverse	CAGAGGTAGATGTCCATAGCTGTTGCATAAGCCAC	P5	P5
	Template	$\alpha1^{\rho1(M1+M2)}$	ρ1 wt	PCR 1 + 2
$\beta2^{\Delta218}$-$\rho1$	Forward	SP6	AAAAGGAACATTTTCTTCTTCTTGCTCCAAAC	SP6
	Reverse	CAAGAAGAAGAAAATGTTCCTTTTCAGCTTAAAG	P5	P5
	Template	β2 wt	ρ1 wt	PCR 1 + 2

Construct	Primers & template	PCR 1 (N-terminal)	PCR 2 (C-Terminal)	PCR 3 (assembly)
$\beta2^{\rho1(M1+M2)}$	Forward	SP6	GCGTGAATGCCACTCTCCCTAAAATCCCCTATGTC	SP6
	Reverse	GGATTTTAGGGAGAGTGGCATTCACGCCCGTG	P5	P5
	Template	$\beta2^{\Delta218}$-$\rho1$	$\beta2$ wt	PCR 1 + 2
$\beta2^{EXT}$-$\rho1^{TM+INT}$	Forward	SP6	CTATGTCAAAGCCATTGACATCTACCTCTGGGTCAGC	SP6
	Reverse	CCAGAGGTAGATGTCAATGGCTTTGACATAGGGGATTTTAG	P5	P5
	Template	$\beta2^{\rho1(M1+M2)}$	$\rho1$ wt	PCR 1 + 2
$\alpha1^{\Delta308}$-$\rho1$	Forward	SP6	GTTTGCCACAGTAAACTATCTGACCACTGTGCAGGAGAG	SP6
	Reverse	GCACAGTGGTCAGATAGTTTACTGTGGCAAACTCAATC	P5	P5
	Template	$\alpha1$ wt	$\rho1$ wt	PCR 1 + 2
$\alpha_1^{\rho1(\Delta8\text{-postM3})}$	Forward	SP6	GCAGGAGAGGAAGGATGGCAAAAGCGTGGTTCC	SP6
	Reverse	GCTTTTGCCATCCTTCCTCTCCTGCACAGTGGTC	P5	P5
	Template	$\alpha1^{\Delta308}$-$\rho1$	$\alpha1$ wt	PCR 1 + 2
$\beta2^{\Delta304}$-$\rho1$	Forward	SP6	CGCTTTGGTCAACTACCTGACCACTGTGCAGGAGAG	SP6
	Reverse	CCTGCACAGTGGTCAGGTAGTTGACCAAAGCGTATTCCAG	P5	P5
	Template	$\beta2$ wt	$\rho1$ wt	PCR 1 + 2
$\beta_2^{\rho1(\Delta8\text{-postM3})}$	Forward	SP6	GCAGGAGAGGAAGCGCCAAAAGAAAGCAGCTGAG	SP6
	Reverse	CTTTCTTTTGGCGCTTCCTCTCCTGCACAGTGGTC	P5	P5
	Template	$\beta2^{\Delta304}$-$\rho1$	$\beta2$ wt	PCR 1 + 2

Supplementary Table 4. Primers and templates used for constructing the chimeric receptors

Final Construct	Primers & templates	
$\rho1^{\alpha1(\Delta6\text{-postM3})}$	Forward primer	AGAGGGTATAGGAAGGAACAGAAGCTGCG
	Reverse primer	CTTGGTGAAGTAGTTGACGGCCGCATAC
	Template	ρ1 wt
$\rho1^{\beta2(\text{M1-M2 link})+\alpha1(\Delta6\text{-postM3})}$	Forward primer	CCTCTGCTGCCAGAGTCCCCTTAGGTATCAC
	Reverse primer	CATCATAGTTGATCCAGAAGGACACCCAGG
	Template	$\rho1^{\alpha1(\Delta6\text{-postM3})}$
$\beta2^{\rho1(\text{M1-M2 link})}$	Forward primer	CCGTGCCTGCACGGGTTGCATTAGGAATTAC
	Reverse primer	CTCTGCGGTCAATCCAAAAGGAGACCCAGGAG
	Template	β2 wt
$\beta_2{}^{\rho1(\text{M1-M2 link + }\Delta8\text{-postM3})}$	Forward primer	CCGTGCCTGCACGGGTTGCATTAGGAATTAC
	Reverse primer	CTCTGCGGTCAATCCAAAAGGAGACCCAGGAG
	Template	$\beta_2{}^{\rho1(\Delta8\text{-postM3})}$
$\rho1^{T349K}$	Forward primer	AACTACCTGACCAAGGTGCAGGAG
	Reverse primer	GACGGCCGCATACTCCAGC
	Template	ρ1 wt
$\alpha1^{L300V}$	Forward primer	CACAGTAAACTATTTCACCAAGAGAGG
	Reverse primer	GCAAACTCAATCACAGCTGAGAAA
	Template	α1 wt
$\beta2^{L296V}$	Forward primer	CTTTGGTCAACTACATCTTCTTTGG
	Reverse primer	CGTATTCCAGAACGGCCATAAAG
	Template	β2 wt
$\alpha1^{G258A}$	Forward primer	CAAGAACTGTCTTTGCAGTGACGACT
	Reverse primer	CTGGTACTGACTCTCTGTTGAGCC
	Template	α1 wt
$\beta2^{G254A}$	Forward primer	CACGGGTTGCATTAGCAATTACAACT
	Reverse primer	CAGCAGAGGCATCATAGTTAATCC
	Template	β2 wt
$\alpha1^{G258V}$	Forward primer	CAAGAACTGTCTTTGTAGTGACGACT
	Reverse primer	CTGGTACTGACTCTCTGTTGAGCC
	Template	α1 wt
$\beta2^{G254V}$	Forward primer	CACGGGTTGCATTAGTAATTACAACT
	Reverse primer	CAGCAGAGGCATCATAGTTAATCC
	Template	β2 wt
$\alpha1^{V296L}$	Forward primer	CTATGCCTTTCTTTTCTCAGCTC
	Reverse primer	CATACTGCAATAAACCAGTCCATAG
	Template	α1 wt
$\beta2^{V292L}$	Forward primer	CTTTGTCTTCCTCTTTATGGCC
	Reverse primer	CACCCCATTAGGTACATGTCAATG
	Template	β2 wt
$\alpha1^{V251A}$	Forward primer	CAGAGAGTCAGAACCAGCAAGAAC
	Reverse primer	TTGAGCCAGAAGGAGACTTGG
	Template	α1 wt
$\beta2^{S247A}$	Forward primer	TATGATGCCGCTGCTGCAC
	Reverse primer	GTTAATCCAAAAGGAGACCCAGGAG
	Template	β2 wt
$\alpha1^{V251I}$	Forward primer	CAGAGAGTCAATACCAGCAAGAAC
	Reverse primer	TTGAGCCAGAAGGAGACTTGG
	Template	α1 wt
$\beta2^{S247I}$	Forward primer	TATGATGCCATTGCTGCACGG
	Reverse primer	GTTAATCCAAAAGGAGACCCAGGAG
	Template	β2 wt
$\alpha1^{V251D}$	Forward primer	CAGAGAGTCAGATCCAGCAAGAAC
	Reverse primer	TTGAGCCAGAAGGAGACTTGG
	Template	α1 wt
$\beta2^{S247D}$	Forward primer	TATGATGCCGATGCTGCACG
	Reverse primer	GTTAATCCAAAAGGAGACCCAGGAG
	Template	β2 wt

Final Construct	Primers & templates	
α1^{V251F}	Forward primer	CAGAGAGTCATTTCCAGCAAGAACTG
	Reverse primer	TTGAGCCAGAAGGAGACTTGG
	Template	α1 wt
β2^{S247F}	Forward primer	TATGATGCCTTTGCTGCACG
	Reverse primer	GTTAATCCAAAAGGAGACCCAGGAG
	Template	β2 wt
α1^{N307Q}	Forward primer	CCACAGTACAGTATTTCACCAAGAGAGG
	Reverse primer	CAAACTCAATCAGAGCTGAGAAAAC
	Template	α1 wt
β2^{N303Q}	Forward primer	CTTTGGTCCAGTACATCTTCTTTGG
	Reverse primer	CGTATTCCAGAAGGGCCATAAAG
	Template	β2 wt
α1^{N307S}	Forward primer	CCACAGTATCCTATTTCACCAAGAGAGG
	Reverse primer	CAAACTCAATCAGAGCTGAGAAAAC
	Template	α1 wt
β2^{N303S}	Forward primer	CTTTGGTCTCCTACATCTTCTTTGG
	Reverse primer	CGTATTCCAGAAGGGCCATAAAG
	Template	β2 wt
α1^{N307V}	Forward primer	CCACAGTAGTCTATTTCACCAAGAGAGG
	Reverse primer	CAAACTCAATCAGAGCTGAGAAAAC
	Template	α1 wt
β2^{N303V}	Forward primer	CTTTGGTCGTCTACATCTTCTTTGG
	Reverse primer	CGTATTCCAGAAGGGCCATAAAG
	Template	β2 wt
α1^{N307D}	Forward primer	CCACAGTAGACTATTTCACCAAGAGAGG
	Reverse primer	CAAACTCAATCAGAGCTGAGAAAAC
	Template	α1 wt
β2^{N303D}	Forward primer	CTTTGGTCGACTACATCTTCTTTGG
	Reverse primer	CGTATTCCAGAAGGGCCATAAAG
	Template	β2 wt
γ2L^{V262F}	Forward primer	CTTTTCCTGCCAGAACATCTTTAGG
	Reverse primer	CATCCTTATTGATCCAGAAGGACAC
	Template	γ2L wt
γ2L^{H318V}	Forward primer	CACCCTGGTTTATTTTGTCAGC
	Reverse primer	CCATACTCCACCAAAGCAGAAAAC
	Template	γ2L wt
GlyRα1$^{\rho1(\Delta6\text{-postM3})}$	Forward primer	GAGAGGAAGCTTCGATTTAGGAGGAAGCG
	Reverse primer	CTGCACAGTGGACACAAAGTTGACAGCG
	Template	GlyRα1 wt
GlyRα1^{L298V}	Forward primer	GGCCGTGCTGGAATATGC
	Reverse primer	GAGAACACGAAGAGCAGGCA
	Template	GlyRα1 wt
GlyRα1^{G256V}	Forward primer	GTGTGGGACTGGTCATCACCAC
	Reverse primer	GAGCTGGTGCAGCATCCAT
	Template	GlyRα1 wt
GlyRα1^{V294L}	Forward primer	CCTGCTCTTCCTGTTCTCGG
	Reverse primer	CAAACAGCCATCCAGATGTCAATAG
	Template	GlyRα1 wt
GlyRα1^{A249F}	Forward primer	ATGGATGCTTTCCCAGCTCGTG
	Reverse primer	GTTGATCCAGAAGGAGATCCAG
	Template	GlyRα1 wt
GlyRα1^{N305V}	Forward primer	CCGCTGTCGTCTTTGTGTCC
	Reverse primer	CATATTCCAGCAGGGCCGAG
	Template	GlyRα1 wt

Supplementary Table 5. Primers and templates used for point mutated receptors.
